# Replication Region Analysis Reveals Non-lambdoid Shiga Toxin Converting Bacteriophages

**DOI:** 10.3389/fmicb.2021.640945

**Published:** 2021-03-18

**Authors:** Ann-Katrin Llarena, Marina Aspholm, Kristin O’Sullivan, Grzegorz Wêgrzyn, Toril Lindbäck

**Affiliations:** ^1^Department of Paraclinical Sciences, Faculty of Veterinary Medicine, Norwegian University of Life Sciences, Oslo, Norway; ^2^Department of Molecular Biology, Faculty of Biology, University of Gdañsk, Gdañsk, Poland

**Keywords:** EHEC, bacteriophage lambda, lytic, phage replication, lysogen, Stx phage

## Abstract

Shiga toxin is the major virulence factor of enterohemorrhagic *Escherichia coli* (EHEC), and the gene encoding it is carried within the genome of Shiga toxin-converting phages (Stx phages). Numerous Stx phages have been sequenced to gain a better understanding of their contribution to the virulence potential of EHEC. The Stx phages are classified into the lambdoid phage family based on similarities in lifestyle, gene arrangement, and nucleotide sequence to the lambda phages. This study explores the replication regions of non-lambdoid Stx phages that completely lack the *O* and *P* genes encoding the proteins involved in initiating replication in the lambdoid phage genome. Instead, they carry sequences encoding replication proteins that have not been described earlier, here referred to as *eru* genes (after EHEC phage replication unit genes). This study identified three different types of Eru-phages, where the Eru1-type is carried by the highly pathogenic EHEC strains that caused the Norwegian O103:H25 outbreak in 2006 and the O104:H4 strain that caused the large outbreak in Europe in 2011. We show that Eru1-phages exhibit a less stable lysogenic state than the classical lambdoid Stx phages. As production of phage particles is accompanied by production of Stx toxin, the Eru1-phage could be associated with a high-virulence phenotype of the host EHEC strain. This finding emphasizes the importance of classifying Stx phages according to their replication regions in addition to their Stx-type and could be used to develop a novel strategy to identify highly virulent EHEC strains for improved risk assessment and management.

## Introduction

Enterohemorrhagic *Escherichia coli* (EHEC) is responsible for severe foodborne diarrheal diseases in humans. The first known large outbreak of food-borne disease due to Shiga toxin-producing *E. coli* occurred in the United States in 1982 and was linked to consumption of undercooked contaminated ground beef prepared as hamburgers (Riley et al., 1983). The *E. coli* O157:H7 strain EDL933 that caused this outbreak is currently reference strain for O157:H7 and EHEC (Riley et al., 1983). The major virulence factor of EHEC is the Shiga-toxins (Stx), whose genes are carried by bacteriophages (Loś et al., 2011). Stx are divided into two major families, Stx1 and Stx2, of which the Stx2 group contains the most heterogenic and most potent variants (Fuller et al., 2011; Scheutz et al., 2012). Indeed, the reference strain EDL933 carries an Stx2 phage designated 933W (O’Brien et al., 1989; Plunkett et al., 1999).

Since 1982, the world has experienced multiple outbreaks of enterohemorrhagic disease caused by other serotypes than O157:H7, of which serogroups O26, O103, O111, and O145 appear to be the most frequent (Mellmann et al., 2008). However, new variants of Shiga toxin-producing *E. coli* are constantly emerging and, in 2011, an EAEC of serotype O104:H4 caused an extensive, multinational outbreak in Europe (Bielaszewska et al., 2011; Rasko et al., 2011). To better understand the pathogenesis of EHEC, the genomes of numerous Stx phages carried by EHEC isolates have been sequenced and compared (Gamage et al., 2004; Ahmed et al., 2012; Beutin et al., 2012; L’Abée-Lund et al., 2012; Smith et al., 2012; Yin et al., 2015). Yin et al. (2015) examined single-nucleotide polymorphisms (SNPs) and insertional elements in the genomes of Stx2aphages from *E. coli* O157:H7, and based on similarities, the phages were classified into three distinct Phage Sequence Types designated PST1, PST2, and PST3. The O157:H7 PST2 phages clustered together with Stx2a phages in non-O157 Shiga-toxin-producing *E. coli* strains associated with a high incidence of hemolytic uremic syndrome (HUS), such as the Norwegian O103:H25 outbreak strain and the German EAEC O104:H4 outbreak strain from 2006 and 2011, respectively (Ahmed et al., 2012; L’Abée-Lund et al., 2012). The PST1 cluster contained phages related to those of the *E. coli* O157:H7 strain Sakai and EDL933, while cluster PST3 contained only the *E. coli* O157:H7 strain EC4115 (Yin et al., 2015). Notably, the Stx2 phage carried by the Norwegian O103:H25 outbreak strain (TL-2011c, JQ011318) has previously been shown to differ from the Stx2 phage 933W of EHEC O157:H7 EDL933 in a 20 kb region of its genome (L’Abée-Lund et al., 2012).

Shiga toxin-converting phages (Stx phages) have until now been regarded as lambdoid bacteriophages. “Lambdoid” is an ambiguously used term and can be defined as phages that are related to and/or are able to recombine with phage lambda to produce replicating progeny. The replication of phage lambda has been studied in depth and the key elements of its replication region are the sequence encoding the repressor CI, the *p*_R_ promoter, and the *O* and *P* genes that encode replication proteins (Casjens and Hendrix, 2015). Phage 933W resembles phage lambda in both nucleotide sequence and gene synteny (O’Brien et al., 1989; Plunkett et al., 1999). They have very similar replication regions including the *O* and *P* genes, which share more than 90% nucleotide sequence identity (Plunkett et al., 1999). The similarity between their replication proteins, especially the O protein, has also been confirmed by functional studies (Kozłowska et al., 2020).

Lambda and lambda-like phages are temperate (lysogenic) viruses; after infection, they follow either a lysogenic pathway which involves integration of phage DNA into the host genome and replication of the phage genetic material along with the chromosome of the host cell or a lytic pathway which leads to cell death and release of new phage particles (Ptashne, 2004; Zeng et al., 2010). The transcriptional regulator CI represses transcription of genes involved in the lytic cycle pathway and is thereby responsible for maintaining the lysogenic state (Kaiser, 1957; Bednarz et al., 2014; Casjens and Hendrix, 2015). Lysogenic phages may, however, switch to a lytic life cycle in response to environmental stresses by inactivating CI (Ptashne, 2004; Wegrzyn and Wegrzyn, 2005). Like phage lambda, the Stx phages act as silent prophages in the lysogenic state (Krüger and Lucchesi, 2015) but can convert into the lytic state under stress conditions, ultimately resulting in cell lysis, release of phage particles, and Shiga toxin (Schmidt, 2001; Loś et al., 2011; Licznerska et al., 2016). Therefore, the regulation of the Stx phage replication cycle significantly influences the level of Stx produced by EHEC and is important for development of enterohemorrhagic disease and HUS in humans (Nejman-Faleńczyk et al., 2012; Nowicki et al., 2013; Balasubramanian et al., 2019).

In the Norwegian outbreak in 2006, caused by the strain EHEC O103:H25 NOS carrying the Stx phage TL-2011c, there was a remarkable high incidence of severe disease and 60% of the reported outbreak cases developed HUS (*n* = 11 of 17). Previous studies have found this strain to produce a high number of Stx2aphages even in the lack of environmental stressors (Iversen et al., 2015). Regulation of phage replication is highly relevant for the level of Stx toxin produced, but the mechanism governing replication of this specific phage is currently unknown. In this study, we have compared nucleotide sequences of Stx phages accessible in databases and classified the phages according to their respective replication regions. The replication region of lambdoid Stx phages such as 933W has already been thoroughly described and, therefore, this study focuses on three other types of Stx phages with replication regions different from lambdoid phages and from each other. Two of the non-lambdoid phage types only carry genes encoding either Stx2a or Stx2c, while the third type only was found to carry genes encoding either Stx1 or Stx2a. Here we show results suggesting that non-lambdoid Stx phages have a more unstable lysogenic state than the lambdoid Stx phages and that phages with this replication region have been isolated from EHEC strains involved in outbreaks with a high HUS-incident. The results indicate that characterization of the replication region of the Stx phage could potentially be used as a novel strategy to identify highly virulent EHEC strains and with that, improve risk assessment and outbreak management strategies.

## Materials and Methods

### Introduction of Eru

In 2006, Norway experienced a severe EHEC outbreak with a high HUS-incident (60%) caused by *E. coli* O103:H25 str. NIPH-11060424. The strain was sequenced and deposited in DDBJ/EMBL/GenBank under the accession no AGSG01, and the sequence of the Stx2 phage genome (TL-2011c) was deposited under the accession no NC_019442. In this study, we examined the nucleotide sequence of TL-2011c more thoroughly to seek answers for the high HUS-incident caused by this particular strain. The most striking observation was that TL-2011c lack genes encoding the replication proteins *O* and *P* of phage lambda. As genomes of most phages are modular and location of genes strongly suggests their functions, we suggest that three genes in the TL-2011c genome appearing at the location corresponding to the replication genes of lambda are involved in replication and are hereafter named *eru1A* (EHEC phage replication unit gene), *eru1B*, and *eru1C* (Table 1). Stx phages carrying the three *eru1* genes are dubbed Eru1-phages.

**TABLE 1:** Description of genes belonging to the three different Eru type phages.

	Seq accession no	Locus tag	Protein id	Description	Phage type
*eru1A*	NC_019442.1	F366_gp30	YP_007001450.1	DEAD/DEAH box helicase	Eru1
*eru1B*	NC_019442.1	F366_gp31	YP_007001451.1	Toprim domain-containing protein	Eru1
*eru1C*	NC_019442.1	F366_gp32	YP_007001452.1	DUF1367 family protein	Eru1
*eru2A*	NC_013008	ECSP_RS14235	WP_000539354.1	Replication protein	Eru2
*eru2B*	NC_013008	ECSP_RS14230	WP_001248388	AAA family ATPase helicase	Eru2
*eru3A*	CP038349	E4U62_17760	QKA54166.1	DNA replication protein	Eru3
*eru3B*	CP038349	E4U62_17755	QKA54165.1	Helicase DnaB	Eru3
*eru1r*	NC_019442.1	F366_gp27	YP_007001447.1	LexA family transcriptional regulator	Eru1
*eru2r*	NC_013008	ECSP_RS14250	WP_001302016.1	LexA family transcriptional regulator	Eru2
*eru3r*	CP038349	E4U62_17780	QKA54170.1	LexA family transcriptional regulator	Eru3

Further BLASTn searches using *stx* as query sequence revealed two other phage types that also lack the lambdoid *O* and *P* genes but with replication regions different from that of TL-2011c. These Stx phage types were dubbed Eru2- and Eru3-phages (Table 1).

### Bacterial Strains and Phages

The bacterial strains and phages used in this study are listed in Supplementary Table 1. The phage infection experiments were performed using non-virulent versions of three different Stx phages, two of them (TL-2011c and phi3538/95) belonging to the Eru1 group of phages and 933W belonging to lambdoid phages (Yin et al., 2015). The phages TL-2011c, Phi3538/95, and 933W were originally carried by the Norwegian EHEC O103:H25 NOS (Schimmer et al., 2008; L’Abée-Lund et al., 2012), the German O157:H7 strain 3538/95 (Schmidt et al., 1999), and the reference strain *E. coli* O157:H7 strain EDL933, respectively (Riley et al., 1983). The phages used are recombinant versions where *stx_2__*a*_* is replaced by the chloramphenicol resistance gene (*cat*; Schmidt et al., 1999; Gamage et al., 2003; Serra-Moreno et al., 2006). *E. coli* DH5α and *E. coli* C600 were used as a recipient strains for the phages.

### Sequence Analysis

To investigate the prevalence of the replication region of the Stx phage TL-2011c, a remote BLASTn search [BLAST + v. 2.11.0 (Altschul et al., 1990), *e*-value 10e-5, and word size 28] among *E. coli* strains (taxid: 562) and phages with short tails (taxid:10744) present in the NCBI nucleotide collection was performed (O’Leary et al., 2016) using the *eru1A-C* genes from the TL2011-c as query sequence. Assemblies of strains containing *eru1A-C* homologs (sequence coverage > 80%, sequence identity > 80%) were called Eru1-phages. To investigate the prevalence of Eru2 and Eru3 phages, BLASTn searches among *E. coli* strains (taxid: 562) present in the NCBI nucleotide collection were performed using either the Eru2-phage genome of EHEC O157:H7 TW14359 (NC_013008; 2671000–2718900) or the Eru3-phage genome of STEC O157:H7 F8952 (CP038349; 3364421–3415841) as query sequence. Assemblies of strains covering at least the replication region and the *stx* genes were called Eru-phages.

To investigate the distribution of different *stx*-carrying Eru-phages, a database 181 of STEC genome sequences of serotypes O157:H7, O111:NM, O111H8, O111: H11, O104:H4, O104:H21, O104:H26, and O104:H7 were constructed (Supplementary Table 7). The database includes all fully closed STEC genomes available in RefSeq in addition to a few selected strains of O157:H7 (*n* = 92), all contig or scaffolds assemblies available in RefSeq in addition to selected strains of O111:H11 (*n* = 2), O111:H8 (*n* = 9), and O111:NM (*n* = 33). For O104-serotypes, the RefSeq database is heavily biases toward the German outbreak in 2011, so additional assemblies of O104-serotypes were sought from Enterobase (Zhou et al., 2020), resulting in a collection of O104:H21 (*n* = 1), O104:H26 (*n* = 1), O104:H7 (*n* = 10), and O104:H4 (*n* = 33). Mashtree v. 1.1.3 with default settings was used to infer whole genome clustering of the strains in the described database (Katz et al., 2019). The Mashtree was used to control for serotype, identify outgroup strain, and choose a suitable reference strain for the O157:H7 (*n* = 101) and O111:NM (*n* = 43) serotype to build the core SNP phylogenies using Snippy v. 4.6.0^1^ and recombination removal with Gubbins v 2.4.1 (Croucher et al., 2015). The resulting recombination free core SNP alignment was used to construct high-resolution phylogeny with FastTree v. 2.1.9 (generalized time-reversible model) to verify the inheritance pattern of the Eru-phages.

### Preparation of Phage Filtrates

Lysogens of *E. coli* C600 carrying 933WCm, TL-2011cCm, or phi3538/95Cm were taken from fresh plates and grown in Lysogeny Broth (LB) for 20 h at 37°C. The cultures were then centrifuged for 10 min at 3,900 *g* and sterile-filtrated using 0.22 μm filters (Millex-GP, Millipore, Bedford, MA, United States). For phage induction by mitomycin C (MMC), cultures were grown to mid-exponential growth phase (OD_600_ = 0.5), induced by 0.5 μg/ml MMC, and incubated further for 18 h. The phage concentration in the bacteria-free filtrate was determined by plaque assay using *E. coli* DH5α as host strain. In order to remove any colicins, trypsin (Sigma) was added to the phage-filtrate to a final concentration of 0.1 mg/ml followed by 1 h incubation at 37°C (Gordon and O’Brien, 2006).

### Plaque Assay

Plaque assay was used to determine the concentration of infective phage particles in the phage filtrates (Iversen et al., 2015). A volume of 100 μl of phage filtrate was mixed with 900 μl of *E. coli* DH5α culture (OD_600_ 0.3) and 3 ml 0.7% LB agar containing 10 mM CaCl_2_ and poured onto LB agar plates. The plates were incubated overnight at 37°C, and plaques were counted. The phage concentration is given as plaque-forming units/ml (PFU/ml). The plaque assay was performed in four independent biological replicates.

### Lysogenic Infection

The ability of 933WCm, TL-2011cCm, and phi3538/95Cm to infect *E. coli* as lysogens was tested as described by others (Schmidt et al., 1999). *E. coli* C600 was used as host strain at a culture cell density of 1 × 10^7^ CFU/ml and a phage concentration of 1 × 10^6^ PFU/ml, giving a multiplicity of infection (MOI) of 0.1. 100 μl phage filtrate was mixed with 100 μl *E. coli* C600 culture and incubated at 150 rpm agitation for 2 h at 37°C. Subsequently, 100 μl of the mix was plated on LB plates and incubated at 37°C overnight. Colonies growing on LB plates containing 34 μg/ml of chloramphenicol were considered to be lysogens. The lysogenic infection assay was performed in three independent biological replicates. Each biological replicate included two technical replicates to determine cell density and phage concentration for calculate of MOI.

## Results

**FIGURE 1:**
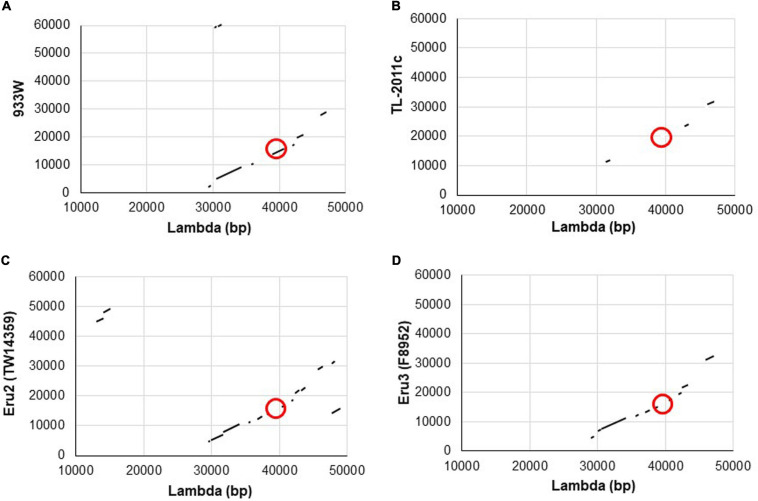
Schematic illustrations of dot matrix views showing nucleotide sequence homology between the genomes of phage lambda and 933W **(A)**, lambda and TL-2011c **(B)**, phage lambda and the Eru2-phage of *E. coli* O157:H7 TW14359 **(C)**, and lambda and the Eru3-phage of *E. coli* O157:H7 F8952 **(D)**. The alignments are based upon BLASTn algorithm and are shown in the plot as line. The query sequence is represented on the *X*-axis, while the subject is represented on the *Y*-axis. The numbers on the *X-* and *Y*-axis represent the bases of the query and subject sequence, respectively. Plus-strand matches are slanted from the bottom left to the upper right corner. The position of the replication regions is marked with red circles.

**FIGURE 2:**
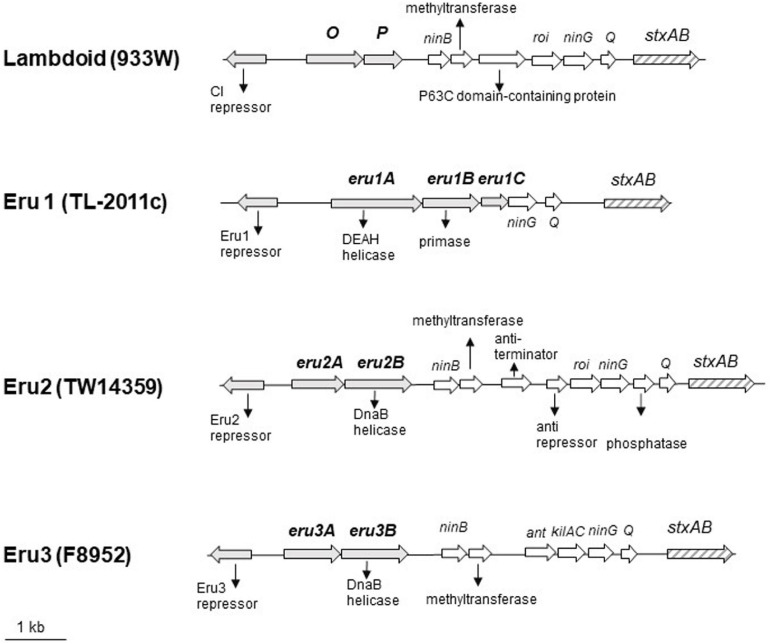
Physical maps of the Stx2 phages carrying the lambdoid (933W, NC_000924; 12075–22702), the Eru1 (TL-2011c, NC_019442; 17619–25677), the Eru2 (EHEC O157:H7 TW14359, NC_013008; 2695183–2707440), and the Eru3 replication region (STEC O157:H7 F8952, CP038349; 3391574–3402867). *eru* genes are indicated by gray arrows, and *stx* genes are indicated by striped arrows.

To explore why the Stx phage TL-2011c produced a high number of phages in the absence of chemical induction (Iversen et al., 2015), we examined the nucleotide sequence of its genome in detail and observed that its replication region differed from the replication region of phage lambda and the Stx phage 933W. A comparison of the nucleotide sequences between 933W (NCBI NC_000924) and phage lambda (NC_001416) showed, as expected, homology over the replication region (red circle, Figure 1A). The overall nucleotide sequence identity between phage TL-2011c (NCBI NC_019442) and phage lambda was, on the other hand, only 4% and they shared no homology in the replication region (Figure 1B). The most striking difference between the two phages is the lack of the genes encoding the replication proteins *O* and *P* in the genome of TL-2011c. Instead, three novel phage replication genes appear at this location; NCBI NC_019442 Gene ID: 14005222, hereafter named *eru1A* (EHEC phage replication unit gene), NCBI NC_019442 Gene ID: 14005223, hereafter named *eru1B*, and NCBI NC_019442 Gene ID 14005224, hereafter named *eru1C* (Figure 2). The Stx phages carrying these genes in their replication region are hereafter dubbed “Eru1-phages.”

Further BLASTn searches using *stx*_2_ as query sequence followed by a manual examination of the upstream replication region revealed two additional phage types, displaying replication regions different from those of 933W and TL2011c. These phage types are hereafter named Eru2- and Eru3-phages and are represented by EHEC O157:H7 TW14359 (NC_013008; 2671000–2718900) and by STEC O157:H7 F8952 (CP038349; 3364421–3415841), respectively. The Eru2- and the Eru3-phages are more similar to phage lambda than the Eru1-phage, with an identity of 98% covering 15% and 97% covering 13% of the lambda genome, respectively (Figures 1C,D). TL-2011c and 933W show 98% identity in the region downstream of the *stx* genes which mainly encode structural proteins (covering 66% of the 933W genome, Supplementary Figure 1A). The Eru1 phage TL-2011c carries genes encoding the Stx2a subtype. The Eru2- and the Eru3-phage showed 99% identity over an area encompassing 40% of the Eru2-phage genome; however, the identity did not cover the *eru* genes (Supplementary Figure 1B). The “structural gene regions” of TL-2011c and the Eru3-phage showed 99% identity (encompassing 48% of the TL-2011c genome, Supplementary Figure 1D), while Eru2 showed less homology to TL-2011c (96% identity covering 11% of the TL-2011c genome, Supplementary Figure 1C). The Eru2-phage of EHEC O157:H7 TW14359 carries genes encoding subtypes Stx2a, and the Eru3-phage of STEC O157:H7 F8952 carries genes encoding the subtype Stx2c. Details of the replication regions of Eru2-and Eru3-phages are shown in Figure 2.

The protein encoded by *eru1A* (Figure 2), Eru1A (YP_007001450.1), consists of 506 amino acids (aa) and belongs to the superfamily 2 (SF2) of helicases. SF2 encompasses one of the largest and most closely related groups of helicases of viral, prokaryotic, and eukaryotic origin (Gorbalenya and Koonin, 1993). Eru1A contains the seven aa motifs, including the DEAH-box (motif II), which are highly conserved among SF2 family proteins (Gorbalenya et al., 1989). The protein encoded by *eru1B* (Figure 2), Eru1B (YP_007001451.1), consists of 323 aa and shares 50–60% sequence identity with the Toprim (topoisomerase-primase) domain-containing proteins of the *Enterobacteriaceae* family. The Toprim domain is a structurally conserved stretch of ∼100 amino acids that is catalytically involved in DNA strand breakage and rejoining (Aravind et al., 1998). The protein encoded by *eru1C* (Figure 2), Eru1C (YP_007001452.1), is a 149 aa homolog of a multispecies *Enterobacreiacea* protein of unknown function (DUF 1367).

The Eru2-phage was first discovered in the EHEC O157:H7 strain TW14359 (NCBI NC_013008), which caused a spinach-associated outbreak in Michigan, United States, in 2006 (Kulasekara et al., 2009; Eppinger et al., 2011). TW14359 carries, in addition to one lambdoid phage encoding Stx2a, an Eru2-phage encoding Stx2c (Fuller et al., 2011; Scheutz et al., 2012). In the Eru2-phage genome, the *O* and *P* genes are replaced by genes dubbed *eru2A* and *eru2B*, respectively, differing from both the lambdoid *O* and *P* genes as well as *eru1A* and *eru1B* of the Eru1 phages (Figure 2). The 273 amino acid protein encoded by *eru2A*, Eru2A (WP_000539354.1), shares 24% *N*-terminal sequence identity with the *O* protein of 933W, mainly over a region containing the Pfam motif ID:Phage_rep_O. The 458-amino acid protein Eru2B (Figure 2; NCBI WP_001248398.1) is distinct from the P protein of 933W (Table 2) and contains two DnaB helicase domains (PF03796 and PF00772) and an ATPase domain PF06745 (GenomeNet Motif Search).

**TABLE 2:** Amino acid sequence identity (%) between replication proteins of Stx phages.

	Eru1A	Eru1B	Eru1r	Eru2A	Eru2B	Eru2r	Eru3A	Eru3B	Eru3r
*O*	**3**	NA	NA	**24**	NA	NA	**3**	NA	NA
*P*	NA	**10**	NA	NA	**12**	NA	NA	**5**	NA
CI	NA	NA	**19**	NA	NA	**11**	NA	NA	**11**
Eru1A	NA	NA	NA	**8**	NA	NA	**1**	NA	NA
Eru1B	NA	NA	NA	NA	**2**	NA	NA	**5**	NA
Eru1r	NA	NA	NA	NA	NA	**47**	NA	NA	**47**
Eru2A	NA	NA	NA	NA	NA	NA	**10**	NA	NA
Eru2B	NA	NA	NA	NA	NA	NA	NA	**32**	NA
Eru2r	NA	NA	NA	NA	NA	NA	NA	NA	**100**

*The given numbers (bold) indicate the allover sequence identity between query protein (first column) and subject protein (upper row). The sequences of the Eru1, Eru2, Eru3, and the lambdoid proteins are derived from the TL-2011c phage, the EHEC O157:H7 strain TW14359, the STEC O157:H7 strain F8952, and the 933W phage, respectively. ^NA^ Not applicable.*

The fourth phage type, Eru3, was first observed in STEC O157:H7 F8952 (CP038349) where *O* and *P* are replaced by *eru3A* and *eru3B*, respectively (Figure 2). The 300 amino acids long proteins encoded by *eru3A and eru3B* [Eru3A (QKA54166.1) and Eru3B (QKA54165.1)] show low amino acid sequence similarity to the O and P proteins of lambdoid phages (Table 2). Eru3B and Eru2B are more similar to each other demonstrating a sequence identity of 31% throughout the proteins. Similar to Eru2B, Eru3B also contains the two DnaB helicase domains PF03796 and PF00772 as well as the ATPase domain PF06745 (GenomeNet Motif Search).

At the location of the gene encoding the CI repressor of 933W (Figure 2), TL-2011c has a gene encoding a putative Eru1-repressor protein (YP_007001447.1) different from the CI protein of 933W (19% identity throughout the aa sequence). However, the putative Eru1-repressor contains the PF00717 domain of peptidase S24 and the PF01381 helix-turn-helix motifs of DNA-binding transcriptional repressors (GenomeNet Motif Search). The Eru1-repressor shows 47% aa identity to the Eru2- and Eru3-repressors, which are identical to each other (Table 2). The aa sequence of the Eru proteins is listed in the Supplementary Material. The DNA sequences of Eru2 and Eru3-phages are more similar to phage lambda in the early gene region than Eru1. Eru2 and Eru3 share identical anti-terminator protein N and CIII, which show 25 and 100% identity to the respective lambda proteins. Cro of Eru1 is 62% identical to the lambda Cro protein, but Eru1 phages lack protein homologs to lambda anti-terminator N, CII, and CIII (Supplementary Table 3).

### Lysogenic Instability of the Eru1-Phages

**FIGURE 3:**
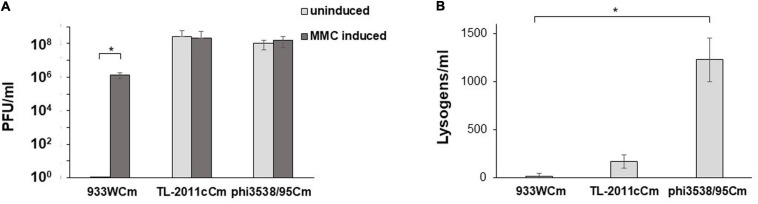
**(A)** Plaque-forming units (PFU/ml) produced by *E. coli* C600 carrying either the lambdoid type 933W phage or the Eru1 type phages TL-2011c or Phi3538/95 with and without MMC induction. **(B)** Lysogens (CFU/ml) at a multiplicity of infection (MOI) of 0.1 in *E. coli* C600. The assays were performed in four **(A)** and three **(B)** independent biological replicates. Error bars represent SD. Asterisks represent statistical differences from pairwise comparisons determined using two-tailed paired Student t tests (^∗^*P* < 0.02).

To elucidate the stability of Eru1-prophages, we compared phage production in *E. coli* C600 with two different Eru1-phages (TL-2011cCm or Phi3538/95Cm) and one lambdoid phage (933WCm) under uninduced and mitomycin C (MMC)-induced conditions. The results indicate that the two Eru1-phages, TL2011 and Phi3538/95, were extremely (eight log) more unstable than the lambdoid phage W933 at uninduced condition (Figure 3A). After 20 h of growth, *E. coli* C600 carrying TL-2011c or Phi3538/95 produce between 1 × 10^7^ and 8 × 10^8^ phage particles regardless of MMC induction, while *E. coli* C600 carrying 933W did not produce any detectable phage particles after 20 h of growth without being exposed to MMC (<1 × 10^1^ PFU/ml). The number of phages produced by *E. coli* C600 carrying 933WCm after induction with MMC was 2 logs lower than for *E. coli* C600 carrying TL2011 or Phi3538/95 (Figure 3A). All three phages can infect *E. coli* C600 and exist as prophages, though Phi3538/95Cm created about ten times more lysogens than TL-2011cCm and 933WCm (Figure 3B). Unfortunately, we did not possess any Eru2Cm- or Eru3Cm-phages to include in the experiment.

### Prevalence of Eru1-Phages

By BLASTn searches using the replication region of TL-2011c (NCBI NC_019442; 17619–23035) as query sequence in the NCBI nucleotide collection of *E. coli* (taxid:562) and phages with short tails (taxid:10744), 101 *E. coli* strains or short tailed phages carrying the Eru1 replication region were identified (Supplementary Table 4). Ninety of the strains were isolated from patients with bloody diarrhea or HUS, and 11 were from fecal samples from livestock. All sequenced isolates from the sprout outbreak in Europe in 2011, the fermented sausage outbreak in Norway in 2006 (L’Abée-Lund et al., 2012), and a restaurant outbreak in United States in 2006 (Eppinger et al., 2011) are represented in Supplementary Table 4 by EHEC O104:H4 German outbreak strain (GOS1), O103:H25 str. NIPH-11060424, and O157:H7 TW14588, respectively. The Eru1-phage sequences were manually checked for the Stx types; however, since the *eru* and the *stx* genes often were split into different contigs, the Stx type of the phages was not always possible to determine. The Eru1-phages carried genes encoding either Stx1 or Stx2a, the latter often associated with severe disease, while some Eru1-phages lacked stx genes completely (Supplementary Table 4). The Eru1-phages were present in a variety of serogroups collected over a wide timespan; EHEC O111:H8 strain DEC8B carrying an Stx phage of the Eru1-type is a clinical isolated from 1986. Notably, Eru1-phages were also found in *Shigella* spp. The *Shigella* phage SS-VASD (NCBI Reference Sequence: NC_028685.1) isolated from two epidemiologically unrelated cases of gastroenteritis in Mexico 2014 (Carter et al., 2016) was very similar to TL-2011c (98% nucleotide identity with a coverage of 90%). The *Shigella sonnei* strain 2015AM-1099 (Sikorski et al., 2020) also carries a prophage similar to TL-2011c although this phage lacks *stx*-encoding genes.

A BLASTn search using the 12 kb sequence covering the replication region and the *stx* genes of the Eru2-phage from EHEC O157:H7 TW14359 (Figure 2) as query sequence in the NCBI RefSeq Genome Database (refseq_genomes) of *E. coli* (taxid:562) revealed 51 additional strains (all of serotype O157:H7), carrying the Eru2-phage (Supplementary Table 5). When possible, we determined the Stx subtype encoded by the Eru2-phages and found that was mainly Stx2c (Supplementary Table 5).

A BLASTn search using the 12 kb sequence covering the replication region and the *stx* genes of the Eru3-phage F8952 (Figure 2) as query sequence in the NCBI RefSeq Genome Database (refseq_genomes) of *E. coli* (taxid:562) revealed 57 additional strains (all of serotype O157:H7), including the Sakai strain, carrying the Eru3-phage (Supplementary Table 6). The nucleotide sequence of the prophage VT2-Sakai of the EHEC O157:H7 derived from the Sakai outbreak (Makino et al., 1999) shows 100% identity with a coverage at 95% to the Eru3-phage F8952. When possible, we determined the Stx subtype encoded by the Eru3-phages and observed that it was mainly Stx2a (Supplementary Table 6).

Enterohemorrhagic *Escherichia coli* strains can carry more than one Stx phage in their genome (Makino et al., 1999; Unkmeir and Schmidt, 2000; Sato et al., 2003; Fogg et al., 2012). In Table 3, we have listed some of the combinations of Stx phages observed among STEC/EHEC O157:H7. We have so far not detected *E. coli* strains carrying both an Eru2 and an Eru3-phage.

**TABLE 3:** Combinations of Stx phages in EHEC/STEC.

		Phage replication type			
Strain	Source				
(accession number)		Lambdoid	Eru1	Eru2	Eru3
O157:H7 EDL 933 (CP008957)	Comminuted beef Outbreak USA 1982	1345558-1356185* (complementary) Stx2a 3012809-3021716* (complementary) Stx1			
O157:H7 Sakai (NC_002695)	Outbreak Japan 1996	2924625-2933532* (complementary) Stx1			1257054-1268347* Stx2a
O157:H7 TW14359 (NC_013008)	Spinach outbreak USA 2006	3225878-3237415* (complementary) Stx2a		2695183-2707440* (complementary) Stx2c	
O157:H7 TW14588 (CM000662)	Restaurant outbreak USA 2006	3900030-3910249* Stx1	3596004-3604062* Stx2a		5564287-5575576* (complementary) Stx2a
O157:H7 NE 1092-2 (CP038328)	Bovine feces Nebraska 2000		1565810-1573577* Stx1	2062966-2075223* Stx2c	

*Nucleotide position from the beginning of the repressor encoding gene to the end of stx genes (Figure 2) in accession number sequence.

### Distribution of Different Types of Stx Phages Between EHEC/STEC Strains

**FIGURE 4:**
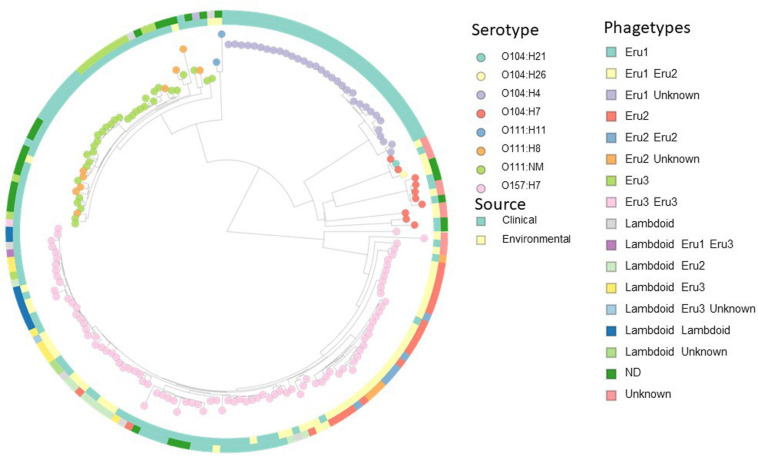
Distance-based cluster analysis with Mashtree based on 181 STEC/EHEC strains (whole genome sequences, scaffolds, and contig assemblies) from serotypes O104:H4, O104:21, O104:H7, O104:H26, O111:H11, O111:NM, O111:H8, and O157:H7. Nodes are colored according to serotype, the inner ring is colored according to source and the outer according to phagetype (see legends). Several strains carry more than one Stx phage, and the combinations of phagetypes are indicated by colors in the outer ring. Eighteen strains carried Stx phages of neither lambdoid, Eru1, 2, nor 3 type (unknown). An interactive version of the figure is available at https://microreact.org/project/2SxKu7mKPw6pumDitTwpn9/8f765953.

The distribution of Eru-phages between *E. coli* strains was investigated to assess the nature of evolution and spread of the Eru-phages. A custom database containing all closed genomes of serotype O157:H7, and all chromosome, scaffolds, and contig assemblies of the serotype O111:H11, O111:NM, O104:H4, O104:21, O104:H7, and O104:H26 available in the RefSeq database (Supplementary Table 7) were subjected to a pairwise all-against-all BLASTn search for homologs of the replication regions (*Eru1A-C, Eru2A-B, Eru3A-B*, and *O-P genes*; Figure 4). After excluding genomes containing Eru-phages without *stx* genes from the resulting list of hits, there were 181 genomes left and, out of these, 64 contained an Eru1-phage (Outer ring, Figure 4). In 59 of the 64 Eru1-phage positive genomes, it was the only Stx phage present. These 59 strains were of serotypes O104:H4, O104:H21, and O157:H7 and all, except two (O157:H7 strains 1125 and 3-5-1) were collected from patients. The five O157:H7 strains containing additional Stx phages to Eru1 were EHEC TW14588, G5295, F6667, NE1092-2, and 95JB1. TW14588, isolated from a restaurant outbreak in United States in 2006, had one lambdoid, one Eru1- and one Eru3-phage. G5295, F6667 and the environmental strain NE1092-2 had both an Eru1- and an Eru2-phage and the clinical strain 95JB1 had an Eru1-phage and an Stx phage that could not be classified as typically lambdoid, Eru1, Eru2, nor Eru3 type.

The incongruent distribution of Eru1 within different serotypes shown in Figure 4 indicates horizontal transmission of the phage. However, the majority of O111:NM, O104:H4, and O157:H7 strains carrying an Eru1-phage clustered together in the WGS analysis (Figure 4 and Supplementary Figures 2, 3), suggesting that vertical transmission of Eru1 also occurs. O104:H4 strains carrying Eru1 clustered monophyletically, and the phage was nearly ubiquitously distributed within this serotype. However, the great majority of the O104:H4 strains (21 out of 32) were linked to the German EHEC outbreak in 2011, and analysis of a more diverse collection is needed to debunk or confirm if Eru1 is native for this serotype. For O111:NM, eight out of 10 strains carrying the Eru1-phage clustered together, which was also the case in the core genome SNP tree (Supplementary Figure 3). The Eru2-phages were restricted to serotype O157:H7 and mainly found in two monophyletic clusters (Figure 4). The Eru3-phages were found in the serotypes O157:H7, O111H8, and O111:NM (Figure 4).

## Discussion

Shiga toxins-producing *E. coli* and *Shigella dysenteriae* type 1 strains can cause severe human disease, but there is still an incomplete understanding of why certain strains appear to be more virulent than others (Unkmeir and Schmidt, 2000; Schmidt, 2001). To explain the variation in virulence observed among EHEC isolates, several studies have compared the phage genomes looking for answers there. However, the herein described differences in the replication region of the Stx phages have, to the best of the authors’ knowledge, never been described before.

In this study, we classify Stx phages according to their respective nucleotide sequence in the replication region and introduce the designation Eru to the phages with a replication region different from that of 933W. All Stx phages have until now been considered lambdoid as the Stx-model phage 933W resembles the model virus of molecular biology—the phage lambda (Loś et al., 2011). The resemblance between lambda and 933W also includes the replication region which determines the life cycle of the phages. The three types of Eru-phages presented in this study carry replication regions different from lambda and 933W as well as from each other. The Eru1*-*phages are distinct from phage lambda, both in the structural and in the replication region, and should possibly not be considered as a lambdoid phage at all. The more important question is, however, how these differences influence the life cycle of the phage and the pathogenic potential of the host EHEC strain.

The lambda CI repressor binds as a dimer to specific operator sites and downregulates the expression of genes involved in production of new phage particles, i.e., the lytic cycle. CI shares sequence similarity and function with the bacterial SOS response master repressor LexA (Schmidt et al., 1999; Serra-Moreno et al., 2006). Upon damage to the hosts DNA, the activated RecA protein proteolytically inactivates the CI repressor (Appleyard, 1954; Hanahan, 1985), and relieved CI repression leads to expression of early and late phage genes and initiation of the lytic cycle. Alternative regulatory mechanisms have been described for several SOS-inducible phages. In *Vibrio cholerae*, the phage CTXΦ, which encodes the cholera toxin, is regulated by the hosts LexA protein and the phage repressor to ensure permanent production and secretion of CTXΦ (Kimsey and Waldor, 2009). Some phages of the viral families *Sipho*- and *Myoviridae* utilize the LexA-regulated antirepressor instead of the cleavable repressor to associate their lytic switch to the host SOS response (Shearwin et al., 1998; Mardanov and Ravin, 2007; Lemire et al., 2011). The repressors of *Salmonella* Gifsy phages do not undergo RecA-mediated proteolysis; they are instead inactivated by complex formation with small antirepressor proteins that causes the repressor to dissociate from DNA. This regulatory system allows separate prophages within a given bacterial strain to be induced simultaneously (Lemire et al., 2011). It has been suggested that such antirepressor-mediated prophage induction is quite common among bacteria (Lemire et al., 2011). The Eru repressor proteins described in the present study likely represent previously undescribed phage repressor proteins that are of high interest for further studies to better understand the varying pathogenic potential of STEC strains.

Shiga toxin exists in two major forms, Stx1 and Stx2, with the Stx2 subtypes a–h. The toxin subtypes are associated with different clinical outcomes, and Stx2a and Stx2d appear to be more potent than Stx1, Stx2b, and Stx2c (Fuller et al., 2011; Scheutz et al., 2012). In this study, we observe that the Eru1-phages can carry genes encoding Stx1 or Stx2a, although some Eru1-phages do not carry *stx* in their genomes at all. Notably, the highly potent Stx2a is encoded by most of the Eru1-phages identified in the present study (Supplementary Table 4). This may, however, be explained by a bias in the sequenced material since highly pathogenic isolates will be sequenced more frequently than the less pathogenic ones. The Eru2-phages are restricted to serotype O157:H7 and are predominant for the less potent Stx2c subtype. Most strains carrying solely Eru2-phages are environmental isolates (Figure 4), which may indicate that this phage has a more limited pathogenic potential. Eru3-phages are carried by both serotype O157:H7 and O111 strains and are more likely to encode the more potent subtype Stx2a. All strains carrying solely Eru3 are clinical isolates (Figure 4), indicating that this phage type may have a more pathogenic potential.

Stx phages infect *E. coli* of different serotypes, and the production level of Shiga toxins can vary substantially between strains (Wagner et al., 1999). Some strains which carry an Eru1-phage are linked to large severe outbreaks of enterohemorrhagic disease, for example, EAEC O104:H4, which caused the German sprout-associated outbreak in 2011 (Bielaszewska et al., 2011; Rasko et al., 2011), EHEC O103:H25 which caused the Norwegian fermented sausage-associated outbreak in 2006 (Schimmer et al., 2008), and EHEC O145:H28, which caused a romaine lettuce-associated outbreak in United States in 2010 (Taylor et al., 2013). The Eru1-phages seem to be related to strains associated with high HUS rates (Yin et al., 2015); the proportion of patients that developed HUS during the German EAEC O104:H4 outbreak was higher than usually seen during outbreaks (22%) and the Norwegian EHEC O103:H25 outbreak was also characterized by an extraordinary high frequency of HUS of 59% (Frank et al., 2011; Grad et al., 2012; L’Abée-Lund et al., 2012). Experiments performed in this study show that *E. coli* C600 carrying either the Eru1-phage TL-2011c or the Eru1-phage phi3538/95 produces considerably more phages under uninduced conditions compared to *E. coli* C600 carrying the lambdoid 933W phage (Figure 3A). As the synthesis of Stx is tightly linked to the lytic state of the Stx phages (Nejman-Faleńczyk et al., 2012; Nowicki et al., 2013; Balasubramanian et al., 2019), we suggest that the unstable lysogenic state of the Eru1-phages could increase the level of toxin released and the virulence potential of the EHEC strains carrying these phages.

Over a 3-month period in France in 1997, 10 children within a small distance of 15 km developed HUS, and the Stx2-producing phage EAEC O111:H2 was isolated from five of the children’s stools (Boudailliez et al., 1997; Morabito et al., 1998). All attempts to isolate Stx2-producing *E. coli* from various food samples were unsuccessful, and a potential person-to-person transmission of EHEC was suggested (Boudailliez et al., 1997). The sequence of the Stx2 phage from this outbreak, Phi191, is highly similar to the Stx2 Eru1-class phage from the German EAEC O104:H4 outbreak strain (Grande et al., 2014; Supplementary Table 4). Also, during the Norwegian EHEC O103:H25 outbreak in 2006, epidemiological investigations suggested foodborne transmission (fermented sausage); however, Stx2 positive EHEC O103:H25 was never isolated from the sausage (L’Abée-Lund et al., 2012). We suggest that the instability of prophages of the Eru1-type could explain why *stx*-positive *E. coli* has never been isolated from the suspected foods; the high frequency of conversion to the lytic state could sometimes eliminate the entire *E. coli* population carrying them.

Forty-five of the 101 Eru1 carrying strains identified in this study are of serotype O157, while the second largest group of 22 isolates carrying an Eru1-phage is O111 (Supplementary Table 4). The oldest sequenced Eru1-phage was from an O111:H8 outbreak of in Texas in 1986 (strain DEC8B; Torres et al., 2005), followed by the French O111:H2 outbreak in 1992 by (strain ED 191; Boudailliez et al., 1997; Morabito et al., 1998).

## Conclusion

The present study suggests that the difference in lysogenic stability between the lambdoid phage and the two Eru1-phages is determined by the replication region, and that the replication region of the phage thereby has an impact of the virulence potential of the host strain. This also corroborates earlier suggestions that efficient DNA replication of Stx phages is crucial for development of EHEC virulence and that the phage replication machinery could be a target for potential anti-EHEC drugs (Nejman-Faleńczyk et al., 2012; Nowicki et al., 2013). The Eru1-phages have been involved in several severe outbreaks of enterohemorrhagic disease with multiple deaths, and therefore appear with a high pathogenic potential. The Eru3-phages mainly encode the potent Stx2a subtype and are often linked to clinical cases and could therefore be considered to exhibit a high pathogenic potential. The Eru2-phages, on the other hand, appear with a less pathogenic potential, encoding the Stx2c subtype and are more rarely linked to clinical cases. The authors recommend further studies to explore if the sequence of the phage replication region can be used as a valuable tool to rapidly assess the virulence and health risks of *E. coli* to improve quality control systems for safe drinking water and food.

## Data Availability Statement

The datasets presented in this study can be found in online repositories. The names of the repository/repositories and accession number(s) can be found in the article/Supplementary Material.

## Author Contributions

TL designed the experiments and wrote the first draft of the manuscript. KO’S and TL performed the experiments. A-KL performed the bioinformatic analyses. A-KL, MA, and GW contributed to data analysis and writing of the manuscript. All authors approved the final manuscript.

## Conflict of Interest

The authors declare that the research was conducted in the absence of any commercial or financial relationships that could be construed as a potential conflict of interest.

## Footnotes

1 https://github.com/tseemann/snippy
