# Supplementary material for: Replication Region Analysis Reveals Non-lambdoid Shiga Toxin Converting Bacteriophages
Source: Front Microbiol. 2021 Mar 18;12:640945. doi: 10.3389/fmicb.2021.640945 (PMC8044961; doi:10.3389/fmicb.2021.640945)
Supplement: Supplementary file 1 [file Presentation_1.pptx]

## Slide 1
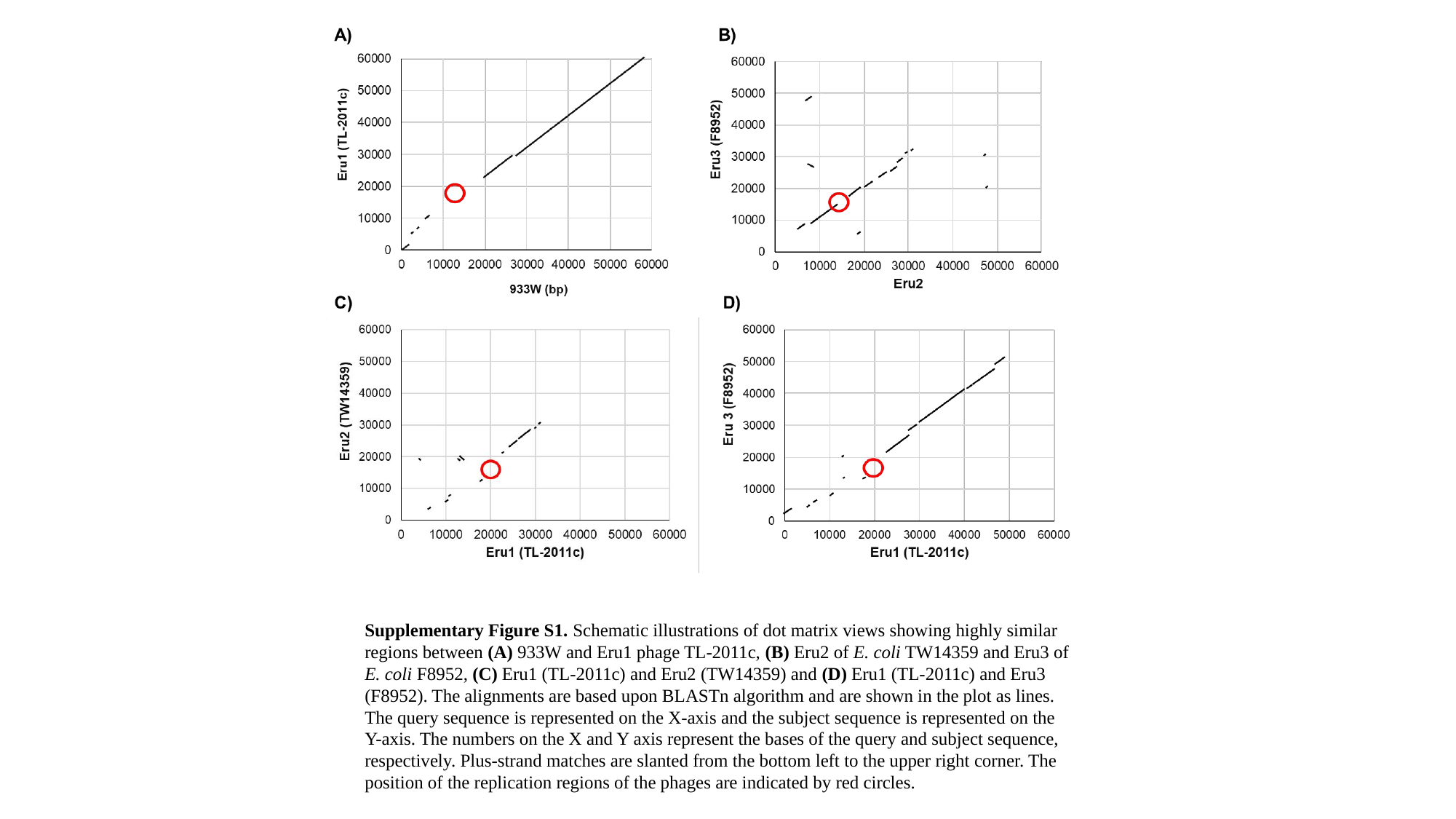

Supplementary Figure S1. Schematic illustrations of dot matrix views showing highly similar regions between (A) 933W and Eru1 phage TL-2011c, (B) Eru2 of E. coli TW14359 and Eru3 of E. coli F8952, (C) Eru1 (TL-2011c) and Eru2 (TW14359) and (D) Eru1 (TL-2011c) and Eru3 (F8952). The alignments are based upon BLASTn algorithm and are shown in the plot as lines. The query sequence is represented on the X-axis and the subject sequence is represented on the Y-axis. The numbers on the X and Y axis represent the bases of the query and subject sequence, respectively. Plus-strand matches are slanted from the bottom left to the upper right corner. The position of the replication regions of the phages are indicated by red circles.
