# Supplementary material for: Replication Region Analysis Reveals Non-lambdoid Shiga Toxin Converting Bacteriophages
Source: Front Microbiol. 2021 Mar 18;12:640945. doi: 10.3389/fmicb.2021.640945 (PMC8044961; doi:10.3389/fmicb.2021.640945)
Supplement: Supplementary file 2 [file Presentation_2.pptx]

## Slide 1
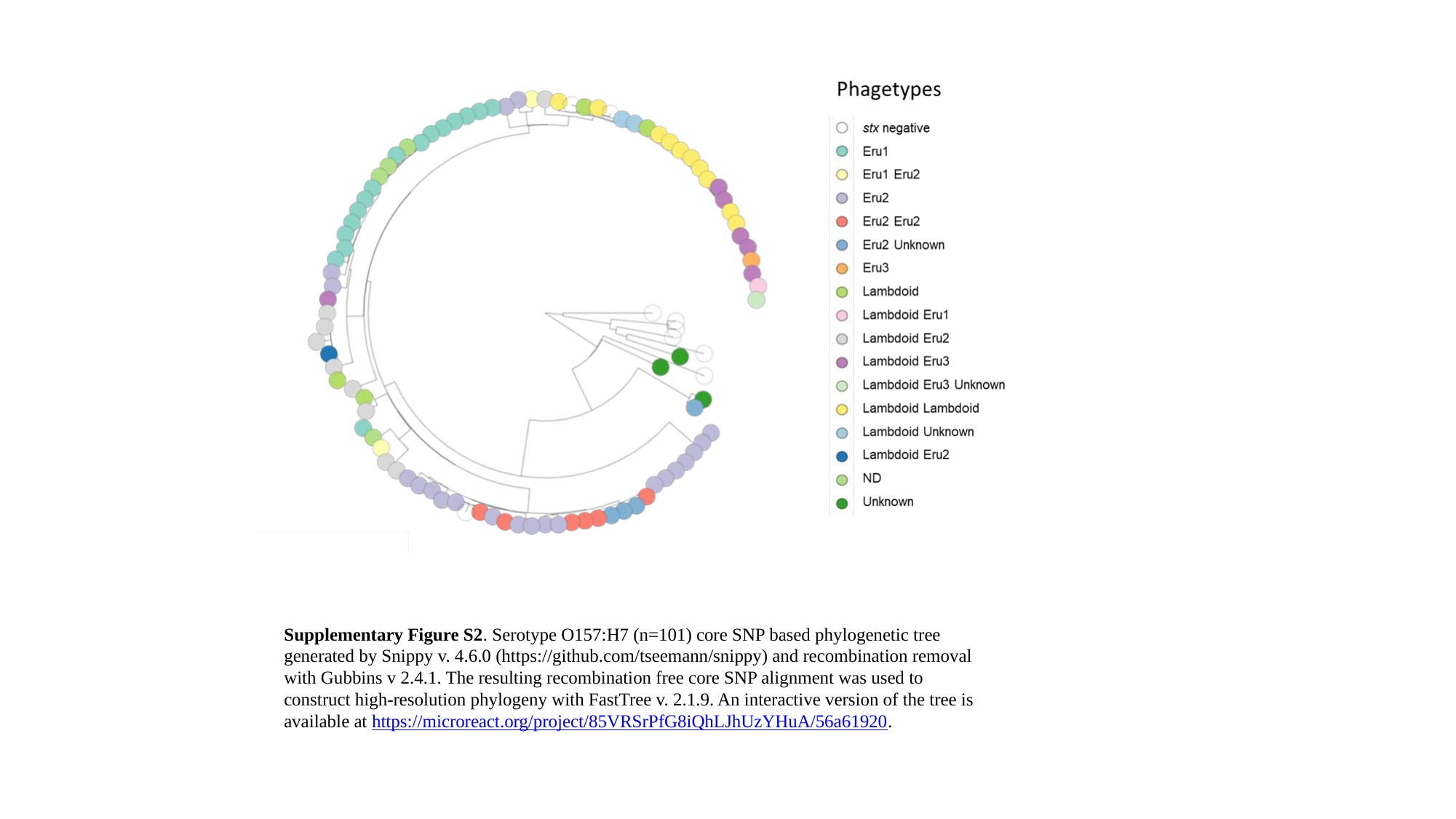

Supplementary Figure S2. Serotype O157:H7 (n=101) core SNP based phylogenetic tree generated by Snippy v. 4.6.0 (https://github.com/tseemann/snippy) and recombination removal with Gubbins v 2.4.1. The resulting recombination free core SNP alignment was used to construct high-resolution phylogeny with FastTree v. 2.1.9. An interactive version of the tree is available at https://microreact.org/project/85VRSrPfG8iQhLJhUzYHuA/56a61920.
