# Supplementary material for: Replication Region Analysis Reveals Non-lambdoid Shiga Toxin Converting Bacteriophages
Source: Front Microbiol. 2021 Mar 18;12:640945. doi: 10.3389/fmicb.2021.640945 (PMC8044961; doi:10.3389/fmicb.2021.640945)
Supplement: Supplementary file 4 [file Table_1.docx]

**Table S1. *E. coli* strains and phages used in this study.**

| **Bacterial strains and phages** | **Characteristics** | **References** | **Accession no**  **NCBI** |
| --- | --- | --- | --- |
| *E. coli* *strains* | | |  |
| C600 | K-12 F*- tonA21 thi-1 thr-1 leuB6 lacY1 glnV44 rfbC1 fhuA1,* λ- | (Appleyard, 1954) |  |
| DH5α | K-12 F^–^ *endA1* *glnV44* *thi-1* *recA1* *relA1* *gyrA96* *deoR* *nupG* *purB20* φ80d*lacZ*ΔM15 Δ(*lacZYA-argF*)U169, hsdR17(*r_K_*^–^*m_K_*^+^), λ^–^ | (Hanahan, 1985) |  |
| *Phages* | | |  |
| TL-2011cCm | Recombinant version of *stx2*-phage from *E. coli* O103:H25, NIPH-11060424, Norwegian outbreak strain from 2006, TL-2011cΔ*stx2*::*cat**. | (Iversen et al., 2015) | NC_019442 |
| phi3538/95Cm | Recombinant version of *stx2*- phage from a patient with HUS, Germany, 1995, phi3538/95Δ*stx2::cat* | (Eichhorn et al., 2018; Schmidt et al., 1999) | DABAXU010000057.1  and  DABAXU010000052.1 |
| 933WCm | Recombinant version of *stx2*- phage from EHEC O157:H7 EDL933, 933WΔ*stx2::cat* | (Gamage et al., 2003) | NC_000924.1 |

* Synonym name of the phage is ϕ734Δ*stx2*::*cat*
