# Supplementary material for: Replication Region Analysis Reveals Non-lambdoid Shiga Toxin Converting Bacteriophages
Source: Front Microbiol. 2021 Mar 18;12:640945. doi: 10.3389/fmicb.2021.640945 (PMC8044961; doi:10.3389/fmicb.2021.640945)
Supplement: Supplementary file 5 [file Table_2.docx]

**Table S2. Amino acid sequence of Eru proteins**

Eru1 from TL-2011c (NC_019442)

Eru1A

>YP_007001450.1 helicase domain protein [Escherichia phage TL-2011c]

MLKITPNFAQERGLNQLRHQWKQHRTYLMYAPTGSGKTGLAAFVTAGMVERGMRVMFVCPYTILLNQTAE

RFTEYGLPWEEISFVWRDHPNYDPSRLIQIASADTLIRREFPDNIDLLIIDEAHMKRRALLEVIRDKDIR

VLGLSGTPFAAWMGKYYECLIKPTTIRELIQRGDLSDYEFFAPSMPDLAGVKTSNTVFGRDYNEEQLASI

MGSSDLVGDIVSNWLENGEDLPTICFCVNVAHANFVTREFLQSGIGAEVMTADTPHDERQDIIRRFEEGA

TKIIVNVGVLVAGFDSDVRCLIYARPTKSEIRWLQCIGRALRTASGKKRALIFDHSGTVHRLGFPEDIEY

DELPGKNDGMKASAGGSEVKAEKLPRECPKCHFMKPAGVHMCPKCGFRPLGGDDVATDRDRKLSRVNKGK

REYTREEKQRWWSEIKGYQNYRNATGKPLSDGWCAHTYKEKFGVWPKGFSNAPLQTSVEVYNFIKSKTIA

YAKGRKKAMTGGQHAN

Eru1B

>YP_007001451.1 putative phage DNA primase [Escherichia phage TL-2011c]

MRTRDAAVGHWSRIFEYYGMPPVTGVKHYNGPCPICGARGKFRCDDKDGSGSWICVCGHGDGMNLLQLAT

GKPWVTLCDEIDRLIGNTWKREKVSQPVTEISRKRELVIDKFAGLPCLRGTTGEAYLQGRGILQLPTESV

RFCDRQIASGREYQAIYAIATDDKGSLCYLHRTLLDGDRKANVEAAKKMTALQELPGLQHAKSVAIRLYP

VSSTLGIAEGIETALSCRQIFRCNVWSTMNSGFMEKFIAPPGVNHLIIFADNDAHGAGLAAAFKCGHKNL

MSCNDVEKVSIRWPDLPDFNDMLIQGCEAREHVLTRKFKAEAA

Eru1C

>YP_007001452.1 hypothetical protein [Escherichia phage TL-2011c]

MEIEMIKAANGVFVPAYERDLPRLAKFKNGELYTLEAKLTRNPSFHRKMFAFLNFCFQYWCAEHAGYEFS

DEATQFDEFRKNLTILAGFYDVVTTIRGEVRYRAKSLSYANMDQDEFERCYNAMINAALKHVFGRSNNPE

LNNRLLSFF

Eru1 repressor

>YP_007001447.1 repressor protein CI [Escherichia phage TL-2011c]

MNMKKKPLTPEQLEDAKRLKSIFNAKKKELGLSQESLAYELGVTQSAVNQLMAGINAINASHAAQLAKIL

NVKVGDFSPSLAKSIAEMALAIEEPLTRVPAYEYPLLSCVQAGAFSMDDISYTAKDAIKWISTTTKASDR

SFWLEVKGHSMTAPQGGKPSFPEGMLILVDPEREIEDGDFCVARMNGDEFTFKRFIRESGKAYLEPLNPR

FDMIECNENCQFVGKVIKSQWNDETFD

Eru2 from EHEC O157:H7 TW14359 (NC_013008)

Eru2A

>WP_000539354.1 MULTISPECIES: replication protein [Escherichia]

MGVVKLADYRPQLEVVEHRVADTEDGFMRVANEITDSLLMADLTVRQLKVMLAIMRKTYGFNKPMDRLTN

TQIAAMTGIHHTHVCAAKRQLIERKFLIADGVKIGVNKVVSQWISQDSLTLAKTANKTLAKSANGYKPSQ

LNTKDNIQKTINTNTPLPPNGDGDGQVKPERRKAERIDYESFLNAYNTEVGDRLPHAVAVNEKRKRRLKK

IIPQLKTPNVDGFRAYVRAFVHQAKPFYFGDNDTGWTADFDYLLREDSLTGVREGKFADRGIA

Eru2B

>WP_001248398.1 MULTISPECIES: AAA family ATPase [Enterobacteriaceae]

MRQDIEASVIGGLLIGGLTPTASDVLATLEPEAFSIPLYRKAFEVIRKQARNRNLIDALMVAEECGEEHF

TSILMTSKNCPSAANLKGYAGMVADNYHRRLVLEIMDEMREPIQSGTIDASSQAMDELVKRLSAIRKPRD

EVKPVRLGEIITDYTDTLDRRLRNGEESDTLKTGIEELDAITGGMNAEDLVIIAARPGMGKTELALKIAE

GVASRVIPGSDVRRGVLIFSMEMSALQIAERSIANAGRMSVSVLRNPASMDDEGWARVANGMSQLADLDV

WVVDASRLSVEEIRSIAERHKQENPNLSLIMADYLGLIEKPKADRNDLAIAHISGSLKAMAKDLKTPVIS

LSQLSRDVEKRPNKRPTNADLRDSGSIEQDADSIIMLYREAVYDENSSAAPFAEIIVTKNRFGSLGTVYQ

RFCNGHFVACDQDEARQICTASNAPAARGRRYAQGADV

Eru2 repressor

>WP_001302016.1 MULTISPECIES: LexA family transcriptional regulator [Enterobacteriaceae]

MDGSSTERNNKDMKMKWYELARSRMKELGITQEKLAEELGMTQGGIGHWLRGSRHPSLSDIGVVFKYLGI

DNISFNHDGTFSPVGEYSSAPVKKQYEYPVFSHVQAGMFSPELRTFTKGDAERLVSTTKKASDSAFWLEV

EGNSMTAPTGSKPSFPDGMLILVDPEQAVEPGDFCIARLGGDEFTFKKLIRDSGQVFLQPLNPQYPMIPC

NESCSVVGKVIASQWPEETFG

Eru3 from STEC O157:H7 F8952 (CP038349)

Eru3A

>QKA54166.1 DNA replication protein [Escherichia coli O157:H7]

MSNISNLAEAREARRLQQPHQSSGKGYALLHRKIMDVPFYKDAEAAHLWVHLILKAKHTPEYVMTDAGEI

LVGRGKLLGGRNSLAFETGLKPDRVQYLLRKFKKLGMIDWVSHGKFSVFSVEKYDDYQSNFVPADYQQIT

TSKPAIPMPASNTVPADYQQITTDKEYNNIISNTDVLESATADKKSDKKKPSVSCQDVVDAYHEILPEAP

KIRALNDKRKNQIRTFWRKAGVITRQLDGHGFTMQDWRNYLSYVGENCRWMFEERPNHQRGTVWHKKGFD

FLLNDNTYLKVREGEHDDR

Eru3B

>QKA54165.1 helicase DnaB [Escherichia coli O157:H7]

MTDNFYAPPHSIEAEQAVIGGLLLDDDSSERVQKVLAMLKPDSFYSRPHKIIFEEITRMHREQKPVDGLT

LFDELERKSLTASVGGFAYIAEIAKNTPSAANIVAYAMQVRETAMERYAINRMTEATELLYSRNGMTATQ

KYEAIQAIFTQLTDHAKTGSRRGLRSFGEVMEDWVSDLEKRFDPSGEQRGMSTGIPSLDRMLSPKGLVKG

SLFVIGARPKMGKTTLYSQMAINCAVHEKKPALMFSLEMPGDQILEKLVGQKSGVNPNIFYLPATNDADD

GYQGDYDGDFNRAIETANRLSEIDLLYIDDTPGLSLAQIVSESRRIKREKGCVGMILVDYLTLMTAEKAD

RNDLAYGMITKGLKNLAKELDCVVVLLTQLNRALESRTNKRPLPSDSRDTGQIEQDCDYWVGIHREGAFD

DSVPPGETELILRLNRHGNTGTVYCIQANGAIYDTDQQSAEMRRREREEPQSKKKGGF

Eru3 repressor

>QKA54170.1 LexA family transcriptional regulator [Escherichia coli O157:H7]

MDGSSTERNNKDMKMKWYELARSRMKELGITQEKLAEELGMTQGGIGHWLRGSRHPSLSDIGVVFKYLGI

DNISFNHDGTFSPVGEYSSAPVKKQYEYPVFSHVQAGMFSPELRTFTKGDAERLVSTTKKASDSAFWLEV

EGNSMTAPTGSKPSFPDGMLILVDPEQAVEPGDFCIARLGGDEFTFKKLIRDSGQVFLQPLNPQYPMIPC

NESCSVVGKVIASQWPEETFG
