# Supplementary material for: Replication Region Analysis Reveals Non-lambdoid Shiga Toxin Converting Bacteriophages
Source: Front Microbiol. 2021 Mar 18;12:640945. doi: 10.3389/fmicb.2021.640945 (PMC8044961; doi:10.3389/fmicb.2021.640945)
Supplement: Supplementary file 6 [file Table_3.docx]

**Table S3.** **% identity between phage regulation proteins of lambda and the respective proteins encoded by the Eru phages**

|  | Eru1 | Eru2 | Eru3 |
| --- | --- | --- | --- |
| Lambda Cro | 62 | 27 | 27 |
| Lammbda CII | 0 | 35 | 35 |
| Lambda CIII | - | 100 | 100 |
| Lambda N | - | 25 | 25 |
