# Supplementary material for: Replication Region Analysis Reveals Non-lambdoid Shiga Toxin Converting Bacteriophages
Source: Front Microbiol. 2021 Mar 18;12:640945. doi: 10.3389/fmicb.2021.640945 (PMC8044961; doi:10.3389/fmicb.2021.640945)
Supplement: Supplementary file 7 [file Table_4.docx]

**Table S4. *E. coli* strains carrying an Eru1 prophage.** The source is human unless otherwise is stated. Not determined (ND) refers to the cases where *eru1* and *stx* genes were split into different contigs.

| **Serotype strain** | **Origin** | **Year** | **Source** | **Accession number** | **Stx- type** |
| --- | --- | --- | --- | --- | --- |
| O157:H7 TW10119 | Germany | 1990 |  | AKMA00000000 | Stx2a |
| O157:H7 TW07945 | USA | 1998 |  | AKLU01000000 | Stx2a |
| O157:H7 B114 | USA | 1999 |  | AVSA00000000 | Stx2a |
| O157:H7 B113 | USA | 1999 |  | AVRZ01000000 | Stx2a |
| O157:H7 B112 | USA | 1999 |  | AVRY01000000 | Stx2a |
| O157:H7 99.1762 | USA | 1999 |  | AOER00000000 | Stx2a |
| O157:H7 99.1793 | USA | 1999 |  | AOEF00000000 | ND |
| O157:H7 99.1805 | USA | 1999 |  | AOEG00000000 | Stx2a |
| O157:H7 Bd5610_99 | Sweden | 1999 | Bovine feces | AVRL00000000 | Neg |
| O157:H7 97.1742 | USA | 1999 |  | ANMA01000000 | Stx2a |
| O157:H7 B28 | USA | 2000 |  | AVQW01000010 | Stx2a |
| O157:H7 B7-2 | USA | 2000 |  | AVRD00000000 | Stx2a |
| O157:H7 B29 | USA | 2000 |  | AVQY00000000 | Stx2a |
| O157:H7 B36 | USA | 2001 |  | AVRA00000000 | Stx2a |
| O157:H7 TW09098 | USA | 2003 |  | AKLX01000000 | ND |
| O157:H7 B90 | USA | 2003 |  | AVSM01000001 | Stx2a |
| O157:H7 B89 | USA | 2003 |  | AVSL00000000 | Stx2a |
| O157:H7 3.4880 | USA | 2003 | Bovine feces | AOET00000000 | ND |
| O157:H7 B93 | USA | 2005 |  | AVRE00000000 | ND |
| O157:H7 06-3745 | USA | 2006 |  | JHNI00000000 | Stx2a |
| O157:H7 TW14313 | USA | 2006 |  | AKMD01000000 | ND |
| O157:H7 PA2 | USA | 2006 |  | AOEL00000000 | ND |
| O157:H7 PA10 | USA | 2007 |  | AKLF01000437 | ND |
| O157:H7 PA9 | USA | 2007 |  | AKLE01000372 | ND |
| O157:H7 ECP17-46 | USA |  |  | CP040572 | Stx2a |
| O157:H7 3-5-1 | USA | 1999 | Bovine feces | CP038416 | Stx2a |
| O157:H7 NE 1092-2 | USA | 2000 | Bovine feces | CP038328 | Stx1 |
| O157:H7 F6667 | USA |  |  | CP038366 | Stx2a |
| O157:H7 PA8 | USA | 2007 |  | AOEO00000000 | Stx2a |
| O157:H7 PA39 | USA | 2008 |  | AKLP01000000 | ND |
| O157:H7 PA34 | USA | 2008 |  | AMTR01000000 | ND |
| O157:H7 8.2524 | USA | 2008 |  | AMTJ01000000 | ND |
| O157:H7 EC1737 | USA | 2009 |  | AMUT01000000 | ND |
| O157:H7 EC1736 | USA | 2009 |  | AMUS01000000 | ND |
| O157:H7 EC1735 | USA | 2009 |  | AMUR01000000 | ND |
| O157:H7 EC1734 | USA | 2009 |  | AKMO01000000 | Stx2a |
| O157:H7 TW14588 | USA | 2006 |  | ABKY02000000 | Stx2a |
| O157:H7 F6142 | USA |  |  | JHJT01000000 | Stx2a |
| O157:H7 EC508 | USA |  |  | ABHW01000000 | Stx2a |
| O157:H7 str. 1125 | USA |  |  | AERR01000000 | Stx2a |
| O157:H7 K1420 | USA |  |  | JHJF01000000 | Neg |
| O157:H7 K4396 | USA |  |  | JHIO00000000 | ND |
| O157:H7 strain 71074 (VT2phi_272) | Canada |  |  | NC_028656* | Stx2a |
| O157:H7 strain F765 (Stx2a_F765) | Japan | 2011 |  | AP012534* | Stx2a |
| O157:H7 strain 3538/95 | Germany | 1995 |  | DABAXU010000000* | Cm |
| O111:H8 DEC8B | USA | 1986 |  | AIGG01000000 | Stx2a |
| O111:H- JB1-95 | USA | 1995 |  | AEZV02000000 | Stx2a |
| O111:H- 95JB1 | Australia | 1995 |  | AWFJ01000000 | Stx2a |
| O111:H- 95NR1 | Australia | 1995 |  | AVDU00000000 | ND |
| O111:H- ATCC BAA-2209 | USA | 2001 |  | AZBZ01000000 | ND |
| O111:H- str. 08-4487 | USA | 2008 |  | JHKU01000000 | Stx1 |
| OK1180 | USA | 2008 |  | ADUQ01000000 | Stx2a |
| O111:H- str. 2009C-4052 | USA | 2009 |  | JHGS01000000 | Stx1 |
| O111:H- str. 2010C-4735 | USA | 2010 |  | JHMU01000000 | ND |
| O111:H- str. 2010C-4715 | USA | 2010 |  | JHMW01000000 | ND |
| O111:H- str. 2011C-3632 | USA | 2011 |  | JHLO01000000 | ND |
| O111:H- str. 2011C-3573 | USA | 2011 |  | JHLQ01000000 | Neg |
| O111:H- str. K6915 | USA |  |  | JHHL01000000 | ND |
| O111:H- str. K6898 | USA |  |  | JHHO01000000 | Stx2a |
| O111:H- str. K6904 | USA |  |  | JHHN01000000 | Stx1 |
| O111:H- str. K6890 | USA |  |  | JHHR01000000 | Stx2a |
| O111:H- str. K6897 | USA |  |  | JHHP01000000 | ND |
| O111:H- str. K6723 | USA |  |  | JHHT00000000 | ND |
| O111:H- str. K6722 | USA |  |  | JHHU01000000 | Stx2a |
| O111:H- str. K6908 | USA |  |  | JHHM01000000 | Neg |
| O111:H- str. K6728 | USA |  |  | JHHS01000000 | Neg |
| O111:H2 str. ED 191 (Phi191) | France | 1992 |  | NC_028660 * | Stx2a |
| O104:H4 str. 09-7901 | France | 2009 |  | AFRK01000000 | Stx2a |
| O104:H4 str. 2009EL-2050/2071 | Republic of Georgia | 2009 |  | CP003297 | Stx2a |
| O104:H4 GOS | Germany | 2011 |  | AFWO00000000 | Stx2a |
| O104:H4 strain ON-2011 (PhiON 2011) | Canada |  |  | KU298437 * | Stx2a |
| O104:H4 strain CB8983 | Germany | 2001 |  | HG792103 | Stx2a |
| O26:H11 ATCC BAA-2196 | USA | 2003 |  | AYOF01000000 | ND |
| O26 str. RM10386 | USA | 2009 | Bovine feces | CP028126 | Stx2a |
| O26:H11 str. 2010C-4819 | USA | 2010 |  | JHMP00000000 | ND |
| O26:H11 str. 2010C-3902 | USA | 2010 |  | JHFH01000000 | ND |
| O103:H2 str. 12009 | Japan | 2001 |  | AP010958 | Stx2a |
| O103:H25 str. NIPH-11060424 (TL-2011c) | Norway | 2006 |  | NC_019442* | Stx2a |
| O145:H- str. 06-3484 | USA | 2006 |  | JHNN01000000 | Stx2a |
| O145:H28 str. RM12581 | USA | 2010 |  | CP007136 | Stx2a |
| O145:H- str. 2010C-3516 | USA | 2010 |  | JHFR01000000 | Stx2a |
| O145:H- (ArgO145) | Argentine |  | Bovine feces | NC_049918.1* | Stx2a |
| O2:H27 (P13803) | Germany | 2001 | Bovine feces | HG792102* | Stx2a |
| O11:H- 99.0741 | USA |  | Bovine feces | AEZR02000000 | ND |
| O43 str. RM10042 | USA | 2009 | Bovine feces | CP028122 | Stx1 |
| O91:H- str. 2009C-3745 | USA | 2009 |  | JHGW00000000 | ND |
| O128:H2 str. 2011C-3317 | USA | 2011 |  | JASU01000000 | Stx1 |
| O149:H? UMNK88 | USA | 2007 | Porcine feces | NC_017641 | Neg |
| O165:H25 str. 2010C-4874 | USA | 2010 |  | JHMM01000010 | ND |
| STEC_EH250 | Belgium | 1995 |  | AFDW01000000 | ND |
| NCCP15648 | South Korea | 2001 |  | CP009050 | Stx2a |
| KTE75 | Denmark | 2010 |  | ANUO01000000 | ND |
| B94 | UK | 2012 |  | AVRF00000000 | Stx2a |
| KTE14 | USA |  |  | ASTU01000000 | Neg |
| C842_97 | Japan | 1997 | Bovine feces | AIBY01000000 | Neg |
| OK1114 | USA | 1989 |  | AICG01000000 | ND |

*The accession numbers refer to the genome sequence of the phage
