# Supplementary material for: Replication Region Analysis Reveals Non-lambdoid Shiga Toxin Converting Bacteriophages
Source: Front Microbiol. 2021 Mar 18;12:640945. doi: 10.3389/fmicb.2021.640945 (PMC8044961; doi:10.3389/fmicb.2021.640945)
Supplement: Supplementary file 8 [file Table_5.docx]

**Table S5. BLASTn hits for Eru2 phages**

| **NCBI nucleotide accession no.** | ***E. coli* strains carrying Eru2 phages (n=52)** | **Stx**  **type** | **Source** |
| --- | --- | --- | --- |
| CP001368.1 | O157:H7 str. TW14359, complete genome | Stx2c | clinical |
| CP034801.1 | O157:H7 strain 2010C-3142 chromosome, complete genome | NA | clinical |
| CP001164.1 | O157:H7 str. EC4115, complete genome | Stx2c | environmental |
| CP034792.1 | O157:H7 strain 2009C-3378 chromosome, complete genome | NA | ND |
| CP038353.1 | O157:H7 strain F8797 chromosome, complete genome | Stx2c | ND |
| CP035545.1 | O157:H7 strain FSIS11705876 chromosome, complete genome | Stx2c | environmental |
| CP046527.1 | O157:H7 strain RM19259 chromosome, complete genome | Stx2c | environmental |
| CP010304.1 | O157:H7 str. SS52, complete genome | Stx2c | ND |
| CP008805.1 | O157:H7 str. SS17, complete genome | Stx2c | ND |
| CP034936.1 | PNUSAE013304 chromosome, complete genome | NA | ND |
| CP033605.1 | O157:H7 strain TR01 chromosome, complete genome | Stx2c | environmental |
| CP014314.1 | O157:H7 strain JEONG-1266, complete genome | Stx2c | environmental |
| CP034799.1 | O157:H7 2009C-4687 chromosome, complete genome | NA | clinical |
| CP030767.1 | O157:H7 2017C-4109 chromosome, complete genome | NA | clinical |
| CP038328.1 | O157:H7 strain NE 1092-2 chromosome, complete genome | Stx2c | environmental |
| CP038414.1 | O157:H7 strain 17B6-2 chromosome, complete genome | Stx2c | environmental |
| CP018252.2 | O157:H7 strain 9000 chromosome, complete genome | Stx2c | environmental |
| CP043015.1 | 388808_gen chromosome, complete genome | NA | clinical |
| CP043025.1 | 399730_gen chromosome, complete genome | NA | clinical |
| CP015853.1 | O157:H7 strain ATCC 43889 chromosome, complete genome | Stx2c | clinical |
| CP043019.1 | 397404_gen chromosome, complete genome | NA | clinical |
| CP043011.1 | 388755_gen chromosome, complete genome | NA | clinical |
| CP034808.1 | O157:H7 strain 08-3914 chromosome, complete genome | NA | clinical |
| CP043539.1 | O157 strain Al Ain chromosome, complete genome | Stx2c | environmental |
| CP018250.1 | O157:H7 strain 10671, complete genome | Stx2c | environmental |
| CP038319.1 | O157:H7 strain NE122 chromosome, complete genome | Stx2c | environmental |
| CP015020.1 | O157:H7 strain 28RC1, complete genome | Stx2c | environmental |
| CP038366.1 | O157:H7 strain F6667 chromosome, complete genome | Stx2c | ND |
| CP022050.2 | O157 strain FDAARGOS_293 chromosome, complete genome | Stx2c | clinical |
| JHKR01000053.1 | O157:H7 str. 2011EL-2103 contig53, whole genome shotgun sequence | Stx2c | clinical |
| AERQ01000009.1 | O157:H7 str. EC1212 isolate A ECD.Contig142_1, whole genome shotgun sequence | Stx2c | environmental |
| ABHL02000004.1 | O157:H7 str. EC4045 gcontig_1113126024295, whole genome shotgun sequence | Stx2c | environmental |
| AKMN01000043.1 | O157:H7 EC1738 EC1738.contig.42, whole genome shotgun sequence | Stx2c | clinical |
| AKLI01000054.1 | O157:H7 PA22 PA22.contig.53, whole genome shotgun sequence | Stx2c | clinical |
| ABHM02000001.1 | O157:H7 str. EC4042 gcontig_1113125281292, whole genome shotgun sequence | Stx2c | clinical |
| ABHK02000001.1 | O157:H7 str. EC4206 gcontig_1113126024111, whole genome shotgun sequence | Stx2c | environmental |
| SEKQ01000043.1 | O157:H7 strain PNUSAE012259 PNUSAE012259_NODE_43_length_15118_cov_54.429725, whole genome shotgun sequence | ND | clinical |
| QSMX02000009.1 | RM10024 unitig_0-quiver, whole genome shotgun sequence | Stx2c | environmental |
| VIFP01000005.1 | 421157, whole genome shotgun sequence | Stx2c | clinical |
| AKLY01000052.1 | O157:H7 TW09109 TW09109.contig.51, whole genome shotgun sequence | Stx2c | clinical |
| AKMA01000035.1 | O157:H7 TW10119 TW10119.contig.34, whole genome shotgun sequence | Stx2c | clinical |
| AKLV01000030.1 | O157:H7 TW10246 TW10246.contig.29, whole genome shotgun sequence | Stx2c | clinical |
| CP034794.1 | O157:H7 06-3462 chromosome, complete genome | Stx2c | clinical |
| ABHP01000075.1 | O157:H7 str. EC4113 gcontig_1105762719101, whole genome shotgun sequence | Stx2c | environmental |
| QSMT02000007.1 | RM10649 unitig_6-quiver, whole genome shotgun sequence | Stx2c | environmental |
| QSMY02000010.1 | RM10718 unitig_10-quiver, whole genome shotgun sequence | Stx2c | environmental |
| QSMW02000009.1 | RM10641 unitig_4-quiver, whole genome shotgun sequence | Stx2c | environmental |
| QSMS02000001.1 | RM10716 unitig_1-quiver, whole genome shotgun sequence | Stx2c | environmental |
| QSMQ02000004.1 | RM10720 unitig_1-quiver, whole genome shotgun sequence | Stx2c | environmental |
| QSMR02000002.1 | RM10719 unitig_0-quiver, whole genome shotgun sequence | Stx2c | environmental |
| QSMV02000001.1 | RM10645 unitig_0-quiver, whole genome shotgun sequence | Stx2c | environmental |
| AVRG01000033.1 | O157:H7 B95 gecB95.contig.32, whole genome shotgun sequence | ND | clinical |

ND: Not determined

NA: Not annotated
