# Supplementary material for: Replication Region Analysis Reveals Non-lambdoid Shiga Toxin Converting Bacteriophages
Source: Front Microbiol. 2021 Mar 18;12:640945. doi: 10.3389/fmicb.2021.640945 (PMC8044961; doi:10.3389/fmicb.2021.640945)
Supplement: Supplementary file 9 [file Table_6.docx]

**Table S6. BLASTn hits for Eru3 phages**

| **NCBI nucleotide accession no.** | ***E. coli* strains carrying Eru3 phages (n=57)** | **Stx**  **type** | **Source** |
| --- | --- | --- | --- |
| CP038349.1 | O157:H7 strain F8952 chromosome, complete genome | Stx2a | clinical |
| CP038342.1 | O157:H7 strain H2495 chromosome, complete genome | Stx2a | clinical |
| CP034803.1 | O157:H7 strain 2009C-3554 chromosome, complete genome | NA | ND |
| CP017444.1 | O157:H7 strain 8368, complete genome | Stx2a | environmental |
| CP038372.1 | O157:H7 strain F6294 chromosome, complete genome | Stx2a | environmental |
| BA000007.3 | O157:H7 str. Sakai DNA, complete genome | Stx2a | clinical |
| CP017669.1 | O157:H7 strain PA20, complete genome | Stx2a | clinical |
| CP062160.1 | O157:H7 strain 20R2R chromosome, complete genome | Stx2a | culture mutant |
| CP028693.1 | O157:H7 strain 108 chromosome, complete genome | Stx2a | environmental |
| CP028607.1 | O157:H7strain 143 chromosome, complete genome | Stx2a | environmental |
| CP040570.1 | O157:H7 strain ECP17-1298 chromosome, complete genome | Stx2a | clinical |
| CP028698.1 | O157:H7 strain 106 chromosome, complete genome | Stx2a | environmental |
| CP016755.1 | O157:H7 strain FORC_044, complete genome | Stx2a | clinical |
| CP017440.1 | O157:H7 strain 3384, complete genome | Stx2a | environmental |
| CP028650.1 | O157:H7 strain 124 chromosome, complete genome | Stx2a | environmental |
| CP044145.1 | O157 strain AR-0428 chromosome, complete genome | NA | ND |
| CP015023.1 | O157:H7 strain SRCC 1675, complete genome | Stx2a | environmental |
| CP064167.1 | O157:H7 strain Wll001 chromosome, complete genome | Stx2a | ND |
| CP041747.1 | O157:H7 strain NCCP 15739 chromosome, complete genome | Stx2a | clinical |
| CP044140.1 | O157 strain AR-0430 chromosome, complete genome | NA | ND |
| CP028609.1 | O157:H7 strain 142 chromosome, complete genome | Stx2a | environmental |
| CP028695.1 | O157:H7 strain 107 chromosome, complete genome | Stx2a | environmental |
| CP028690.1 | O157:H7 strain 109 chromosome, complete genome | Stx2a | environmental |
| CP028687.1 | O157:H7 strain 110 chromosome, complete genome | Stx2a | environmental |
| CP028652.1 | O157:H7 strain 123 chromosome, complete genome | Stx2a | environmental |
| CP028700.1 | O157:H7 strain 105 chromosome, complete genome | Stx2a | environmental |
| JHLH01000065.1 | O157:H7 str. 2011EL-2091 contig65 | Stx2a | clinical |
| JHLD01000034.1 | O157:H7 str. 2011EL-2096 contig34 | Stx2a | clinical |
| JHLG01000002.1 | O157:H7 str. 2011EL-2092 contig2 | Stx2a | clinical |
| JHLC01000032.1 | O157:H7 str. 2011EL-2097 contig32 | Stx2a | clinical |
| JHLF01000009.1 | O157:H7 str. 2011EL-2093 contig9 | Stx2a | clinical |
| JHLI01000009.1 | O157:H7 str. 2011EL-2090 contig9 | Stx2a | clinical |
| JHLB01000035.1 | O157:H7 str. 2011EL-2098 contig35 | Stx2a | clinical |
| JHLE01000042.1 | O157:H7 str. 2011EL-2094 contig42 | Stx2a | clinical |
| JH705566.1 | PA32 PA32.contig.555 | Stx2a | clinical |
| AERP01000008.1 | O157:H7 str. 1044 isolate A ECA.Contig245_1 | Stx2a | environmental |
| SDVX01000006.1 | O157:H7 strain C1-158 Seq6 | Stx2a | environmental |
| RROI01000045.1 | O157:H7 strain FWSEC0413 FWSEC0413_contig00047 | Stx2a | environmental |
| BIAN01000010.1 | 35-1BK entry10 | Stx2a | environmental |
| JACBNR010000029.1 | O157:H7 strain ZZb1 scaffold29.1 | Stx2a | environmental |
| JACBNU010000031.1 | O157:H7 strain ZZb4 scaffold31.1 | Stx2a | environmental |
| JACBNS010000027.1 | O157:H7 strain ZZb2 scaffold27.1 | Stx2a | environmental |
| RICC01000006.1 | O157:H7 strain C1-067 Seq6 | Stx2a | environmental |
| JACBNT010000028.1 | O157:H7 strain ZZb3 scaffold28.1 | Stx2a | environmental |
| AOTB01000001.1 | O157:H7 str. EC1825 Contig1 | Stx2a | clinical |
| PDED01000001.1 | O157:H7 strain FDAARGOS_398 pRIID_102.contig.0, whole genome shotgun sequence | Stx2a | clinical |
| RZTY01000002.1 | O157:H7 strain 2009C-4207 contig2_2009C-4207 | Stx2a | clinical |
| JHKT01000024.1 | O157:H7 str. 2011EL-2099 contig24 | Stx2a | clinical |
| QGWI01000159.1 | O157:H7 ECI-2503 ECI-2503_contig159 | Stx2a | clinical |
| CM000662.1 | O157:H7 str. TW14588 chromosome | Stx2a | clinical |
| ABHT01000068.1 | O157:H7 str. EC4501 gcontig_1106627450961 | Stx2a | clinical |
| QGZB01000054.1 | O157:H7 strain ECI-1159 ECI-1159_contig54, whole genome shotgun sequence | ND | clinical |
| NWON01000052.1 | O157:H7 strain MOD1-EC4337 MOD1-EC4337_52_length_17339_cov_55.8917 | Stx2a | environmental |
| QGWM01000094.1 | O157:H7 strain ECI-2406 ECI-2406_contig94 | Stx2a | clinical |
| PDEK01000002.1 | O157:H7 strain FDAARGOS_399 pRIID_103.contig.1 | Stx2a | clinical |
| PDDT01000002.1 | O157:H7 strain FDAARGOS_400 pRIID_104.contig.1 | Stx2a | clinical |
| QGVR01000146.1 | O157:H7strain ECI-2527 ECI-2527_contig146 | Stx2a | clinical |

ND: Not determined

NA: Not annotated
