# Supplementary material for: Replication Region Analysis Reveals Non-lambdoid Shiga Toxin Converting Bacteriophages
Source: Front Microbiol. 2021 Mar 18;12:640945. doi: 10.3389/fmicb.2021.640945 (PMC8044961; doi:10.3389/fmicb.2021.640945)
Supplement: Supplementary file 10 [file Table_7.docx]

**Table S7. Strains included in Figure 4.**

| id | Name | Serotype | Source | Phage-types |
| --- | --- | --- | --- | --- |
| GCA_000353545.1 | E92/11 | O104:H4 | clinical | Eru1 |
| ESC_BA0018AA_AS | OLC-816 | O104:H7 | environmental/other | Unknown |
| ESC_FA5995AA_AS | PNUSAE001030 | O104:H4 | clinical | Eru1 |
| ESC_GA4741AA_AS_genomic | 94-3024 | O104:H21 | clinical | Unknown |
| ESC_GA4778AA_AS_genomic | RM9387 | O104:H7 | environmental/other | Unknown |
| ESC_HA7896AA_AS | MOD1-EC6922 | O104:H26 | environmental/other | ND |
| ESC_HA8652AA_AS | MOD1-EC2594 | O104:H7 | environmental/other | Unknown |
| ESC_HA8685AA_AS | MOD1-EC2868 | O104:H7 | clinical | ND |
| ESC_HA8687AA_AS | MOD1-EC2870 | O104:H7 | clinical | Unknown |
| ESC_HA8688AA_AS | MOD1-EC2871 | O104:H7 | clinical | ND |
| ESC_HA8689AA_AS | MOD1-EC2872 | O104:H7 | clinical | ND |
| ESC_OA7579AA_AS | FDAARGOS_402 | O104:H4 | clinical | Eru1 |
| ESC_PA5905AA_AS | OLC2136 | O104:H7 | environmental/other | Unknown |
| ESC_VA2307AA_AS_genomic | FDAARGOS_401 | O104:H4 | clinical | Eru1 |
| ESC_VA2308AA_AS_genomic | FDAARGOS_403 | O104:H4 | clinical | Eru1 |
| ESC_WA7718AA_AS | FWSEC0278 | O104:H7 | environmental/other | ND |
| ESC_WA9254AA_AS | FWSEC0087 | O104:H7 | environmental/other | Unknown |
| GCF_000008865.2_ASM886v2 | Sakai | O157:H7 | clinical | Lambdoid_Eru3 |
| GCF_000021125.1_ASM2112v1 | EC4115 | O157:H7 | clinical | Lambdoid_Eru2 |
| GCF_000022225.1_ASM2222v1 | TW14359 | O157:H7 | clinical | Lambdoid_Eru2 |
| GCF_000155125.1_ASM15512v1 | TW14588 | O157:H7 | clinical | Lambdoid_Eru1_Eru3 |
| GCF_000172055.1_ASM17205v1 | EC508 | O157:H7 | clinical | Eru1 |
| GCF_000188835.1_ASM18883v2 | OK1180 | O111:NM | clinical | Eru1 |
| GCF_000192685.1_ASM19268v2 | 1125 | O157:H7 | environmental/other | Eru1 |
| GCF_000194355.1_ASM19435v2 | JB1-95 | O111:NM | clinical | ND |
| GCF_000217695.1 | TY-2482 | O104:H4 | clinical | Eru1 |
| GCF_000217975.1 | H112180280 | O104:H4 | clinical | Eru1 |
| GCF_000222505.1 | H112180282 | O104:H4 | clinical | Eru1 |
| GCF_000223015.1 | GOS1 | O104:H4 | clinical | Eru1 |
| GCF_000223035.1 | GOS2 | O104:H4 | clinical | Eru1 |
| GCF_000235125.1 | 11-4632 C1 | O104:H4 | clinical | Eru1 |
| GCF_000235145.1 | 11_4623 | O104:H4 | clinical | Eru1 |
| GCF_000235165.1 | 11_4522 | O104:H4 | clinical | Eru1 |
| GCF_000235185.1 | 11_4404 | O104:H4 | clinical | Eru1 |
| GCF_000235205.1 | 11_3677 | O104:H4 | clinical | Eru1 |
| GCF_000235225.1 | 09_7901 | O104:H4 | clinical | Eru1 |
| GCF_000235245.1 | 04_8351 | O104:H4 | clinical | Eru1 |
| GCF_000235265.1 | C236-11 | O104:H4 | clinical | Eru1 |
| GCF_000235285.1 | C227-11 | O104:H4 | clinical | Eru1 |
| GCF_000258615.1 | ON2011 | O104:H4 | clinical | Eru1 |
| GCF_000267045.1_ASM26704v1 | EC1734 | O157:H7 | clinical | Eru1 |
| GCF_000267185.1_ASM26718v1 | TW10119 | O157:H7 | clinical | Eru1 |
| GCF_000267285.2_ASM26728v2 | EC4436 | O157:H7 | clinical | Eru1 |
| GCF_000267625.2_ASM26762v2 | TW07945 | O157:H7 | clinical | Eru1 |
| GCF_000276745.1_EC_O111_H8_CVM9602_v01 | CVM9602 | O111:H8 | clinical | ND |
| GCF_000276765.1_EC_O111_H8_CVM9634_1.0 | CVM9634 | O111:H8 | environmental/other | ND |
| GCF_000276865.1_EC_O111_CVM9455_1.0 | CVM9455 | O111:H11 | environmental/other | ND |
| GCF_000276925.1_EC_O111_H11_CVM9553_1.0 | CVM9553 | O111:H11 | environmental/other | Lambdoid |
| GCF_000299255.1 | 2009EL-2050 | O104:H4 | clinical | Eru1 |
| GCF_000299475.1 | 2009EL-2071 | O104:H4 | clinical | Eru1 |
| GCF_000313445.1_CFSAN001632_1.0 | CFSAN001632 | O111:H8 | clinical | ND |
| GCF_000316725.2_ASM31672v2 | 97.1742 | O157:H7 | clinical | ND |
| GCF_000319975.1 | Ec11-4987 | O104:H4 | clinical | Eru1 |
| GCF_000320045.1 | Ec11-9941 | O104:H4 | clinical | Eru1 |
| GCF_000320075.1 | 11-02093 | O104:H4 | clinical | Eru1 |
| GCF_000320195.1 | Ec11-4988 | O104:H4 | clinical | Eru1 |
| GCF_000320215.1 | Ec11-5603 | O104:H4 | clinical | Eru1 |
| GCF_000320255.1 | Ec12-0465 | O104:H4 | clinical | Eru1 |
| GCF_000320275.1 | Ec12-0466 | O104:H4 | clinical | Eru1 |
| GCF_000335035.2_ASM33503v2 | 99.1805 | O157:H7 | clinical | ND |
| GCF_000335155.2_ASM33515v2 | 99.1762 | O157:H7 | clinical | ND |
| GCF_000335455.2_ASM33545v2 | PA8 | O157:H7 | clinical | ND |
| GCF_000350005.1 | E112/10 | O104:H4 | clinical | Eru1 |
| GCF_000462105.2_ASM46210v2 | B29-2 | O157:H7 | clinical | Eru1 |
| GCF_000462405.2_ASM46240v2 | B7-2 | O157:H7 | clinical | Eru1 |
| GCF_000462605.2_ASM46260v2 | B112 | O157:H7 | clinical | Eru1 |
| GCF_000462625.2_ASM46262v2 | B113 | O157:H7 | clinical | Eru1 |
| GCF_000462645.2_ASM46264v2 | B114 | O157:H7 | clinical | Eru1 |
| GCF_000462865.2_ASM46286v2 | B90 | O157:H7 | clinical | Eru1 |
| GCF_000477495.2_ASM47749v2 | B28-2 | O157:H7 | clinical | Eru1 |
| GCF_000615575.2_Ec2010C-3977 | 2010C-3977 | O111:NM | clinical | Eru3 |
| GCF_000615605.2_Ec2010C-4086 | 2010C-4086 | O111:NM | clinical | ND |
| GCF_000615655.2_Ec2010C-4221 | 2010C-4221 | O111:NM | clinical | Eru3 |
| GCF_000616195.2_Ec2011C-3362 | 2011C-3362 | O111:NM | clinical | Lambdoid |
| GCF_000616305.2_Ec2011C-3632 | 2011C-3632 | O111:NM | clinical | ND |
| GCF_000616325.2_Ec2011C-3679 | 2011C-3679 | O111:NM | clinical | Eru3 |
| GCF_000616385.2 | 2011EL-1675A | O104:H4 | clinical | Eru1 |
| GCF_000616685.2_Ec08-4487 | Ec08-4487 | O111:NM | clinical | Eru1 |
| GCF_000617025.2_Ec2010C-4715 | 2010C-4715 | O111:NM | clinical | ND |
| GCF_000617245.2_Ec03-3484 | Ec03-3484 | O111:NM | clinical | Eru3 |
| GCF_000617265.1_Ec04-3211 | Ec04-3211 | O111:NM | clinical | Eru3 |
| GCF_000617465.2_Ec2010C-4735 | 2010C-4735 | O111:NM | clinical | Eru1 |
| GCF_000617485.2_Ec2010C-4746 | 2010C-4746 | O111:NM | clinical | ND |
| GCF_000617525.2_Ec2010C-4799 | 2010C-4799 | O111:NM | clinical | Eru3 |
| GCF_000617545.2_Ec2010C-4818 | 2010C-4818 | O111:NM | clinical | Eru3 |
| GCF_000617625.2_Ec2010C-4622 | 2010C-4622 | O111:NM | clinical | Eru3 |
| GCF_000617805.2_Ec2010C-4592 | 2010C-4592 | O111:NM | clinical | ND |
| GCF_000618125.1_EcF6627 | F6627 | O111:H8 | clinical | ND |
| GCF_000619005.2_Ec2009C-4052 | 2009C-4052 | O111:NM | clinical | Eru1 |
| GCF_000619225.2_Ec2009C-4006 | 2009C-4006 | O111:NM | clinical | ND |
| GCF_000619465.2_EcK6722 | K6722 | O111:NM | clinical | Eru1 |
| GCF_000619485.1_EcK6723 | K6723 | O111:NM | clinical | Eru1 |
| GCF_000619525.1_EcK6890 | K6890 | O111:NM | clinical | Eru1 |
| GCF_000619545.2_EcK6895 | K6895 | O111:NM | clinical | Eru3 |
| GCF_000619585.2_EcK6898 | K6898 | O111:NM | clinical | Eru1 |
| GCF_000619625.1_EcK6904 | K6904 | O111:NM | clinical | Eru1 |
| GCF_000619645.2_EcK6915 | K6915 | O111:NM | clinical | Eru1 |
| GCF_000632595.2_Ec2009EL-2169 | 2009EL-2169 | O111:H8 | clinical | ND |
| GCF_000632615.2_Ec2010C-3053 | 2010C-3053 | O111:NM | clinical | ND |
| GCF_000632635.1_Ec2009C-4126 | 2009C-4126 | O111:H8 | clinical | ND |
| GCF_000632675.2_Ec2011C-3453 | 2011C-3453 | O111:H8 | clinical | ND |
| GCF_000701125.2_ASM70112v2 | 01_3076 | O111:NM | clinical | Eru3 |
| GCF_000730345.1_ASM73034v1 | SS17 | O157:H7 | environmental/other | Lambdoid_Eru2 |
| GCF_000732965.1_ASM73296v1 | EDL933 | O157:H7 | clinical | Lambdoid_Lambdoid |
| GCF_000803705.1_ASM80370v1 | SS52 | O157:H7 | environmental/other | Lambdoid_Eru2 |
| GCF_001307215.1_ASM130721v1 | WS4202 | O157:H7 | clinical | Lambdoid_Unknown |
| GCF_001558995.2_ASM155899v2 | JEONG-1266 | O157:H7 | environmental/other | Lambdoid_Eru2 |
| GCF_001651925.2_ASM165192v2 | FRIK2069 | O157:H7 | environmental/other | Eru2_Unknown |
| GCF_001651945.2_ASM165194v2 | FRIK2533 | O157:H7 | environmental/other | Eru2_Unknown |
| GCF_001651965.2_ASM165196v2 | FRIK2455 | O157:H7 | environmental/other | Eru2_Unknown |
| GCF_001695515.1_ASM169551v1 | FRIK944 | O157:H7 | environmental/other | Eru2_Eru2 |
| GCF_001753445.1_ASM175344v1 | 1130 | O157:H7 | environmental/other | Eru2 |
| GCF_001753465.1_ASM175346v1 | 2149 | O157:H7 | environmental/other | Eru2 |
| GCF_001753485.1_ASM175348v1 | 8368 | O157:H7 | environmental/other | Lambdoid_Eru3 |
| GCF_001753505.1_ASM175350v1 | 2159 | O157:H7 | environmental/other | Eru2 |
| GCF_001753525.1_ASM175352v1 | 9234 | O157:H7 | environmental/other | Eru2 |
| GCF_001753545.1_ASM175354v1 | 3384 | O157:H7 | environmental/other | Lambdoid_Eru3 |
| GCF_001753565.1_ASM175356v1 | 4276 | O157:H7 | environmental/other | Eru2 |
| GCF_002156825.1_ASM215682v1 | 95JB1 | O111:NM | clinical | Eru1_Unknown |
| GCF_002209105.2 | FDAARGOS_348 | O104:H4 | clinical | Eru1 |
| GCF_002983645.1 | FDAARGOS_349 | O104:H4 | clinical | Eru1 |
| GCF_003072445.1 | LB226692 | O104:H4 | clinical | Eru1 |
| GCF_003293905.1_ASM329390v1 | 7-14 10A | O111:H8 | environmental/other | ND |
| GCF_003293935.1_ASM329393v1 | CL-37 | O111:H8 | clinical | Eru3 |
| GCF_003293955.1_ASM329395v1 | 00-4748 | O111:NM | clinical | Eru3 |
| GCF_003294015.1_ASM329401v1 | 3007-85 | O111:NM | clinical | ND |
| GCF_003722195.1_ASM372219v1 | TR01 | O157:H7 | environmental/other | Lambdoid_Eru2 |
| GCF_003966795.1_ASM396679v1 | PV15-279 | O157:H7 | clinical | Eru2_Unknown |
| GCF_005037735.1_ASM503773v2 | FWSEC0004 | O157:H7 | clinical | Lambdoid_Lambdoid |
| GCF_005037805.1_ASM503780v2 | FWSEC0005 | O111:NM | clinical | Eru3_Eru3 |
| GCF_005885915.1_ASM588591v1 | ECP17-1298 | O157:H7 | clinical | Lambdoid_Eru3 |
| GCF_005885955.1_ASM588595v1 | ECP17-46 | O157:H7 | clinical | Eru1 |
| GCF_009650175.1_ASM965017v1 | ATCC43890 | O157:H7 | clinical | Lambdoid |
| GCF_009931235.1_ASM993123v1 | FRIK804 | O157:H7 | environmental/other | Lambdoid_Lambdoid |
| GCF_013167135.1_ASM1316713v1 | F8492 | O157:H7 | environmental/other | Lambdoid_Eru2 |
| GCF_013167155.1_ASM1316715v1 | YB14-1 | O157:H7 | environmental/other | Eru2 |
| GCF_013167175.1_ASM1316717v1 | TX 376-2 | O157:H7 | environmental/other | Eru2 |
| GCF_013167195.1_ASM1316719v1 | TX 265-1 | O157:H7 | environmental/other | Eru2_Eru2 |
| GCF_013167235.1_ASM1316723v1 | TB21-1 | O157:H7 | environmental/other | Eru2 |
| GCF_013167275.1_ASM1316727v1 | SS-tx-754-1 | O157:H7 | environmental/other | Eru2 |
| GCF_013167295.1_ASM1316729v1 | ss-tx-313-1 | O157:H7 | environmental/other | Eru2 |
| GCF_013167315.1_ASM1316731v1 | ss-ne-1040-1 | O157:H7 | environmental/other | Eru2 |
| GCF_013167335.1_ASM1316733v1 | KS470-1 | O157:H7 | environmental/other | Eru2_Eru2 |
| GCF_013167355.1_ASM1316735v1 | OK1 | O157:H7 | clinical | Lambdoid_Lambdoid |
| GCF_013167375.1_ASM1316737v1 | NE92 | O157:H7 | environmental/other | Eru2 |
| GCF_013167395.1_ASM1316739v1 | NE122 | O157:H7 | environmental/other | Eru2 |
| GCF_013167415.1_ASM1316741v1 | NE1127 | O157:H7 | environmental/other | Lambdoid_Lambdoid |
| GCF_013167435.1_ASM1316743v1 | NE1169-1 | O157:H7 | environmental/other | Lambdoid_Lambdoid |
| GCF_013167455.1_ASM1316745v1 | NE1092-2 | O157:H7 | environmental/other | Eru1_Eru2 |
| GCF_013167475.1_ASM1316747v1 | N8B7-2 | O157:H7 | environmental/other | Eru2 |
| GCF_013167515.1_ASM1316751v1 | H6437 | O157:H7 | clinical | Eru1 |
| GCF_013167535.1_ASM1316753v1 | H2495 | O157:H7 | clinical | Lambdoid_Eru3 |
| GCF_013167555.1_ASM1316755v1 | Gim1-1 | O157:H7 | environmental/other | Eru2 |
| GCF_013167575.1_ASM1316757v1 | G5295 | O157:H7 | clinical | Eru1_Eru2 |
| GCF_013167595.1_ASM1316759v1 | F8952 | O157:H7 | clinical | Eru3 |
| GCF_013167615.1_ASM1316761v1 | F8798 | O157:H7 | clinical | Lambdoid_Eru2 |
| GCF_013167635.1_ASM1316763v1 | F8797 | O157:H7 | clinical | Lambdoid_Eru3 |
| GCF_013167655.1_ASM1316765v1 | F8092B | O157:H7 | clinical | Eru2 |
| GCF_013167675.1_ASM1316767v1 | F7508 | O157:H7 | clinical | Lambdoid_Lambdoid |
| GCF_013167695.1_ASM1316769v1 | F7386 | O157:H7 | clinical | Lambdoid |
| GCF_013167715.1_ASM1316771v1 | F7349 | O157:H7 | clinical | Lambdoid_Lambdoid |
| GCF_013167735.1_ASM1316773v1 | F6667 | O157:H7 | clinical | Eru1_Eru2 |
| GCF_013167755.1_ASM1316775v1 | F6321 | O157:H7 | environmental/other | Lambdoid_Eru2 |
| GCF_013167775.1_ASM1316777v1 | F6294 | O157:H7 | environmental/other | Lambdoid_Eru3_Unknown |
| GCF_013167795.1_ASM1316779v1 | F3113 | O157:H7 | environmental/other | Unknown |
| GCF_013167815.1_ASM1316781v1 | F1273 | O157:H7 | environmental/other | Eru2 |
| GCF_013167875.1_ASM1316787v1 | E32511 | O157:H7 | clinical | Eru2 |
| GCF_013168035.1_ASM1316803v1 | DEC4E | O157:H7 | clinical | Lambdoid |
| GCF_013168055.1_ASM1316805v1 | BB24-1 | O157:H7 | environmental/other | Eru2_Eru2 |
| GCF_013168075.1_ASM1316807v1 | ATCC35150 | O157:H7 | clinical | Lambdoid_Unknown |
| GCF_013168095.1_ASM1316809v1 | 86-24 | O157:H7 | clinical | Lambdoid |
| GCF_013168115.1_ASM1316811v1 | 493/89 | O157:H7 | clinical | Unknown |
| GCF_013168135.1_ASM1316813v1 | 17B6-2 | O157:H7 | environmental/other | Eru2 |
| GCF_013168155.1_ASM1316815v1 | ASM1316815v1 | O157:H7 | environmental/other | Eru1 |
| GCF_013168175.1_ASM1316817v1 | ASM1316817 | O157:H7 | environmental/other | Eru2_Eru2 |
| GCF_013168195.1_ASM1316819v1 | 7636 | O157:H7 | environmental/other | Lambdoid_Eru2 |
| GCF_013168215.1_ASM1316821v1 | 7409 | O157:H7 | environmental/other | Eru2 |
| GCF_013168235.1_ASM1316823v1 | 2571 | O157:H7 | environmental/other | Lambdoid_Eru2 |
| GCF_013168255.1_ASM1316825v1 | 611 | O157:H7 | environmental/other | Lambdoid_Eru3 |
| GCF_013343595.1_ASM1334359v1 | MB41-1 | O157:H7 | environmental/other | Eru2 |
| GCF_013343615.1_ASM1334361v1 | USDA5905 | O157:H7 | environmental/other | Unknown |
| GCF_013343635.1_ASM1334363v1 | MB9-1 | O157:H7 | environmental/other | Eru2_Eru2 |
